# A Dusty Affair: SIRT1-S682 Modulation Orchestrates ERK–FN1–p38–NF-κB Signaling and Composite-Dependent IL-8 Responses in Gingival Keratinocytes Exposed to Dental Dust and Eluates

**DOI:** 10.3390/jfb17060264

**Published:** 2026-06-01

**Authors:** Shuoqiu Bai, Sibylle Johanna Rau, Thorsten Steinberg, Pascal Tomakidi, Olga Polydorou

**Affiliations:** 1 Division of Oral Biotechnology, Center for Dental Medicine, Medical Center—University of Freiburg, Faculty of Medicine, University of Freiburg, Hugstetterstr. 55, 79106 Freiburg, Germany; shuoqiu.bai@uniklinik-freiburg.de (S.B.); sibylle.rau@uniklinik-freiburg.de (S.J.R.);; 2 Department of Operative Dentistry and Periodontology, Center for Dental Medicine, Medical Center—University of Freiburg, Medical Faculty, University of Freiburg, Hugstetterstr. 55, 79106 Freiburg, Germany; 3 Department of Conservative Dentistry and Periodontology, Faculty of Medicine, Ulm University, Ulm University Hospital, Albert-Einstein-Allee 11, 89081 Ulm, Germany; olga.polydorou@uniklinik-ulm.de

**Keywords:** dental composite dust, gingival keratinocytes, SIRT1-S682 regulation, NF-κB activation, p38-MAPK, ERK–FN1 axis, particulate cytocompatibility, ormocer-based composite, inflammatory epithelial responses

## Abstract

Dental composite dust generated during finishing procedures or mastication may adversely affect gingival epithelia. However, the mechanistic distinction between particulate and chemical (eluate) exposures and their respective signaling consequences remains insufficiently defined. Dust particles and corresponding eluates from three restorative composites, Admira Fusion, Ceram.x Spectra ST, and Filtek Supreme XTE, were evaluated under standardized high-dose in vitro exposure conditions. Human gingival keratinocytes were assessed for proliferation, adhesion, differentiation, fibronectin (FN1) remodeling, and IL-8 secretion, alongside analysis of ERK, p38, and NF-κB signaling and phosphorylation of the stress-responsive regulator SIRT1 at Ser682 (SIRT1-S682). Particulate exposure elicited more pronounced impairment of cellular adhesion, proliferation, and differentiation than eluates. Dusts derived from Ceram.x Spectra ST and Filtek Supreme XTE suppressed ERK activity, reduced FN1 abundance, and decreased nuclear SIRT1-S682, consistent with a generalized stress response. In contrast, Admira Fusion dust preserved FN1, activated ERK signaling, reduced SIRT1-S682, and induced robust IL-8 secretion. Across all materials, particulate exposure reduced nuclear SIRT1-S682 without affecting total SIRT1 levels, indicating a shared permissive stress modification. Notably, only Admira Fusion coupled this permissive state with p38 activation and sustained NF-κB p65 Ser536 phosphorylation, resulting in transcriptionally active NF-κB and elevated IL-8 production, whereas Ceram.x Spectra ST and Filtek Supreme XTE failed to activate this ERK–FN1–p38–NF-κB axis, yielding either transcriptionally inactive NF-κB or no detectable enrichment. These findings support a material-associated in vitro response pattern in which a shared SIRT1-S682 reduction is accompanied by distinct ERK/FN1, p38, NF-κB, and IL-8 readouts. SIRT1-S682 reduction alone did not define the inflammatory phenotype, because it occurred across particulate exposures, whereas IL-8 secretion was observed only under conditions that also showed p38 activation and comparatively maintained NF-κB p65 Ser536 phosphorylation. This signature arises from the convergence of a permissive SIRT1-S682 background with ERK- and p38-dependent MAPK signaling to enable NF-κB-mediated IL-8 expression, highlighting that both composite composition and particulate properties critically determine inflammatory potential and underscoring the importance of incorporating particulate fractions into cytocompatibility testing strategies.

## 1. Introduction

Dental composites are among the most widely used restorative materials, offering aesthetic, wear-resistant and minimally invasive management of carious lesions. Because billions of resin-based composite restorations are placed worldwide each year [1], it is critical to understand not only their mechanical performance but also their biological interactions within the oral cavity. Advances in resin chemistry, filler technology and polymerization have improved handling and clinical longevity [2]. Nonetheless, mechanical finishing, polishing and chewing release nano- and micro-sized particulate debris. These particles can be inhaled by dental professionals or settle in gingival crevices, directly contacting epithelial cells [3]. While extensive research has focused on leached monomer (eluate) toxicity, the biological consequences of particulate dust remain poorly defined. Distinguishing between physical (particulate) and chemical (eluate) exposures is therefore essential, because they engage different signaling pathways.

Despite these developments, routine clinical procedures such as finishing and polishing inevitably generate nano- to micro-sized particulate debris; chewing can similarly release these particles [2,4]. Beyond occupational exposure concerns for dental professionals [2], these particles accumulate in gingival crevices and directly interact with epithelial tissues. Gingival keratinocytes, which constitute the primary barrier of the oral mucosa, are central to maintaining tissue integrity and orchestrating responses to environmental stressors [5]. In contrast to the extensive body of work addressing cytotoxic effects of eluates derived from incompletely polymerized composites [6,7], the biological consequences of particulate composite dust exposure remain poorly defined. This distinction is critical, as particulate and chemical exposures are mechanistically distinct and are likely to engage divergent cellular signaling pathways. Supporting this notion, inhalable dental dusts have been shown to induce cytotoxic and proinflammatory responses in pulmonary epithelial models [8]. However, whether comparable mechanisms operate in gingival epithelia, and how these differ from eluate-driven effects, remains unclear.

Previous studies have focused primarily on monomeric eluates from resin-based composites [9]. However, mechanical finishing and chewing generate nano- and microparticulate dust that directly contacts gingival epithelia [10,11]. Particulate and chemical exposures activate distinct regulatory axes; conflating them obscures material-specific mechanisms. We sought to establish a conceptual framework that distinguishes general stress responses from composite-specific inflammatory signatures. Central to this framework is the stress-responsive NAD^+^-dependent deacetylase SIRT1. Phosphorylation of SIRT1 at Ser682 disrupts its interaction with the co-activator AROS and inhibits SIRT1 activity [12], suggesting that changes in this phosphorylation site may reflect altered nuclear stress regulation. Importantly, SIRT1-S682 modulation has not previously been integrated into epithelial responses to dental materials. We hypothesized that particulate dust elicits a shared reduction in SIRT1-S682, but that IL-8 production requires additional material-associated signaling events, including ERK/FN1 remodeling, p38 activation, and transcriptionally competent NF-κB signaling. Evidence that fibronectin fragments can activate TLR4-dependent p38/MK2 and NF-κB pathways to regulate IL-8 expression [13] supports this hypothesis. This conceptual advance bridges SIRT1 post-translational regulation with extracellular matrix–derived inflammatory signaling and underscores the need for cytocompatibility testing that incorporates particulate fractions and patient-specific factors such as chewing patterns and salivary pH [11].

Keratinocyte homeostasis depends on tightly regulated processes of proliferation, differentiation, and extracellular matrix (ECM) remodeling. Early differentiation markers such as keratin 1 (KRT1) and keratin 10 (KRT10) reflect the transition from basal to suprabasal states, whereas involucrin (IVL) and filaggrin (FLG) are essential for terminal differentiation and barrier formation [14]. Disruption of these processes compromises epithelial integrity and may predispose tissues to chronic inflammation and impaired wound healing.

The ECM component fibronectin (FN1) plays a central role in keratinocyte adhesion, migration, and tissue repair. FN1 expression and organization are tightly regulated by ERK signaling, which coordinates proliferative and structural responses during epithelial maintenance [15]. Perturbation of ERK activity may therefore directly impact ECM stability and epithelial function.

At the nuclear level, SIRT1 integrates metabolic and stress-related signals to regulate genomic stability, oxidative stress responses, and inflammatory pathways [12]. Its activity is modulated by post-translational modifications, among which phosphorylation at Ser682 has emerged as a negative regulatory mechanism under stress conditions [16]. Consequently, alterations in SIRT1-S682 levels are best interpreted as changes in regulatory state rather than total protein abundance.

Inflammatory signaling in gingival epithelia is critically governed by the NF-κB pathway and its downstream effector CXCL8 (IL-8), a key mediator of neutrophil recruitment and amplification of local inflammation [17]. Importantly, NF-κB activation requires not only nuclear translocation but also phosphorylation of the p65 subunit at Ser536, a modification that enhances transcriptional competence and may be differentially regulated by upstream kinases such as p38 MAPK.

Although recent composite formulations aim to reduce biological reactivity, their effects on gingival epithelial function—particularly under particulate exposure—remain insufficiently characterized. Furthermore, physicochemical properties of particulate debris, including particle size distribution, aggregation behavior, and nanoscale surface morphology, are likely to critically influence cellular responses but have not been systematically evaluated. Integrating morphometric characterization with functional readouts is therefore essential to resolve material-specific determinants of epithelial signaling.

In this study, we systematically investigated the differential effects of composite-derived particulate dust and corresponding eluates on gingival keratinocyte function, including proliferation, differentiation, FN1 regulation, and IL-8–associated inflammatory responses. By integrating ERK–FN1 signaling with SIRT1-S682 as a stress-responsive regulatory node, we aimed to define composite-specific patterns of epithelial perturbation and to delineate mechanistic pathways that distinguish particulate from chemical exposure.

## 2. Materials and Methods

### 2.1. Dental Restorative Materials

Three dental restorative materials—Admira Fusion (VOCO GmbH, Cuxhaven, Germany), Ceram.x^®^ Spectra ST (Dentsply Sirona, Konstanz, Germany) and Filtek™ Supreme XTE (Solventum Corporation, Maplewood, NI, USA)—were prepared according to ISO 7405:2018 and the manufacturers’ instructions. Samples (*n* = 10 per material) were fabricated in molds with a diameter of 8 mm and a height of 2 mm. The composite material was inserted into the mold in one increment and covered with a clear polyester strip before polymerization to prevent oxygen inhibition. Polymerization was performed using an LED light-curing unit Bluephase C8 (Ivoclar Vivadent, Schaan, Liechtenstein) with an intensity of 1250 mW/cm^2^. The tip of the curing unit was placed directly on the polyester strip. All specimens were prepared in accordance with the manufacturers’ instructions for each material. After curing, specimens were disinfected in 70% ethanol for 60 s and air-dried before further processing.

### 2.2. Preparation of Dust and Eluates

Dust Collection: The grinding process was performed in a sealed plexiglass box (50 × 50 × 50 cm) lined with anti-static film to reduce electrostatic interference during dust collection. Each cured specimen was ground using a diamond bur (25 μm; Komet Dental, Lemgo, Germany) operated at 20,000 rpm to generate composite particulate debris. The filtered dust was then collected in sterile cell culture dishes, sealed with plastic wrap, and stored at 4 °C.

Eluate Collection: Eluates were prepared following ISO 10993-12:2012. Briefly, 300 μg of composite dust was suspended in 1 mL of keratinocyte growth medium 2 (KGM2, PromoCell, Heidelberg, Germany), (final concentration, 300 μg/mL, and incubated at 37 °C with continuous shaking. After 72 h, the samples were removed, centrifuged at 10,000× *g* for 30 min to obtain the eluate, and stored at −40 °C until further use. We used a dust concentration of 300 μg/mL (≈94 μg/cm^2^ in a 96-well format) because it lies at the upper range of doses employed in prior in vitro studies of composite dust and environmental particulates. Earlier work exposing human gingival keratinocytes to composite dust tested 3, 10, 30, 100 and 300 μg/mL; fibronectin expression increased with concentration and large agglomerates were observed only at ≥100 μg/mL [18]. A similar concentration series (1–300 μg/mL) was used for lung epithelial cells, where IL-8 release became significant only at 300 μg/mL [19]. Environmental toxicology studies of ultrafine road dust also expose Calu-3 cells to 0–300 μg/mL and report that the highest dose induces maximal reactive oxygen species production and pro-inflammatory cytokine release [20]. Although 300 μg/mL likely exceeds instantaneous intra-oral particle concentrations, it provides a worst-case scenario and enables detection of dose-dependent cellular responses. Because the present study did not include a full dose–response series, the threshold concentration required to induce IL-8 secretion or signaling changes remains unknown. Future studies should include lower and intermediate concentrations, repeated exposure protocols, and clinically informed deposition models to determine whether the signaling patterns observed here occur across a physiologically relevant dose range. Although grinding each specimen in a sealed plexiglass box provides a standardized approach to particle generation, it does not replicate the aerosolization and mechanical attrition occurring during high-speed operative procedures or the prolonged wear and degradation of restorations in situ. Clinical studies show that aerosol-generating procedures such as drilling or denture grinding produce respirable microparticles (<10 μm) that can be inhaled by dental staff and patients [11]. Moreover, polymer-based restorations and prostheses release small particles during simulated mastication and cleaning, and these releases may accumulate in the oral cavity or gastrointestinal tract over months [11]. Real-world exposure patterns are episodic and influenced by patient-specific factors; most micro- and nanoplastic studies indicate that in vitro models do not mirror the dynamic aerosolization and long-term wear experienced clinically [11]. Therefore, our controlled grinding model represents a simplified approximation of clinical conditions, and direct extrapolation to patient exposure should be made cautiously.

### 2.3. Scanning Electron Microscopy (SEM) and Particle Cluster Metrics Analysis

Using SEM, the ultrastructural features and aggregation behavior of composite dust particles adhering to the keratinocyte surface were visualized. After exposure to composite dust, the cells were gently washed with DPBS to remove non-adherent particles and fixed with 4% paraformaldehyde. The samples were dehydrated using an ascending ethanol series (30%, 50%, 70%, 80%, 90%, and 100% ethanol), dried at the critical point, mounted on aluminum stubs, and sputter-coated with a gold-palladium layer for 80 s (~20 nm nominal thickness). SEM imaging was performed using a field emission microscope (JSM 6510, JEOL, Tokyo, Japan) at an acceleration voltage of 10–12 kV and a working distance of 8–10 mm. Images were acquired at 500× or 1000× magnification (10 kV accelerating voltage). For calibration, the 50 μm scale bar in each micrograph (≈500 px) was measured, yielding 0.10 μm px^−1^ (500× images) or 0.05 μm px^−1^ (1000× images). The bottom 12% of each image containing the scale bar was removed prior to analysis.

Images were processed in Python 3.10 (Python Software Foundation (PSF), Wilmington, DE, USA) using the OpenCV and scikit-image libraries. Pre-processing involved conversion to 8-bit greyscale, histogram equalization and adaptive thresholding. Otsu’s method was used to create a binary dust mask, followed by morphological opening and closing (3 × 3-pixel kernel) to remove noise and fill small holes. Connected components were labeled, and clusters smaller than 10 pixels (~0.1 μm^2^) were excluded as debris. For each cluster, the following morphometric parameters were calculated: projected area (μm^2^)—number of pixels within the region multiplied by the squared pixel size. Equivalent circular diameter (μm)—diameter of a circle having the same area as the region. Circularity—4π × (area)/(perimeter^2^), where 1 = perfect circle. Solidity—region area divided by its convex hull area (values near 1 indicate compact, non-concave shapes). Aspect ratio—ratio of major to minor axis lengths from an ellipse fitted to the region. Clusters with circularity ≥ 0.6 were classed as compact; those below this threshold were considered irregular. The number of clusters was normalized to the keratinocyte surface area (determined from a manually defined cell mask) and expressed as particles mm^−2^. All image processing and particle morphometric analyses were performed by the authors in a Python-based environment. The scripts were generated, executed, and verified by the authors and are available upon request for reproducibility.

Statistical analysis for Cluster Metrics: Morphometric data were pooled across replicates for each material. Statistical analyses were performed in Python using SciPy and statsmodels. Normality was assessed with the Shapiro–Wilk test. Because most parameters exhibited non-Gaussian distributions with occasional outliers, values are presented as mean ± standard deviation. One-way analysis of variance (ANOVA) was applied to compare the three materials for each parameter. When significant main effects were found (*p* < 0.05), Tukey’s honestly significant difference (HSD) post hoc test was used for pairwise comparisons. *p*-values < 0.05 were considered significant; in the summary table, different superscript letters denote significant differences.

### 2.4. Cell Culture

The immortalized human gingival keratinocyte cell line (HGKs) used in this study was established in our working group and has been previously described and characterized in detail by Roesch-Ely et al. [21]. The cell line is an established, fully anonymized human-derived cell line and is also commercially available; no primary human cells or newly obtained patient-derived material were used in the present study. At the time of sample collection and cell line establishment, approval was obtained from the Ethics Committee of the Medical Faculty of Heidelberg University, Heidelberg, Germany, on [Approval ID: 148/2003, date 30 September 2005], and written informed consent was obtained from the donor. The original donor material was anonymized, and the present experiments were conducted exclusively with the established cell line. The study was conducted in accordance with the principles of the Declaration of Helsinki. HGKs were cultured in keratinocyte growth medium (KGM2 with supplements and kanamycin) under standard cell culture conditions at 37 °C, 95% humidity, and 5% CO_2_. For particulate exposure, composite dust was suspended directly in fresh KGM2 at a final concentration of 300 μg/mL, and fresh KGM2 without dust served as the medium-only negative control. For eluate exposure, eluates were prepared by incubating composite dust in KGM2 under the same conditions used for the test extracts. The corresponding negative control was process-matched KGM2 that underwent the same incubation, centrifugation, aliquoting, freezing, thawing, and warming steps as the eluate samples, but without composite dust. After 72 h of culture, when HGKs had reached approximately 60–80% confluence, cells were exposed to composite dust suspensions, eluates, or the corresponding exposure-matched controls for 24 h. For experiments involving ERK inhibition, DMSO vehicle controls were included at the same final DMSO concentration as in U0126-treated samples to exclude solvent-related effects. The cells were incubated for another 24 h for subsequent experiments. HPV16 E6/E7-immortalized human gingival keratinocytes (IHGK) because they retain key features of primary gingival keratinocytes, including the ability to form stratified epithelium and express gingival-specific gene products [21]. These cells exhibit a cytokeratin pattern and tight-junction-associated transepithelial electrical resistance comparable to primary keratinocytes, indicating that they can recapitulate epithelial barrier functions [22]. IHGK lines have been routinely used since their characterization nearly 20 years ago [21]. Nevertheless, the presence of E6/E7 oncoproteins could modulate stress-response and inflammatory pathways. For example, E6 and E7 from high-risk HPV types upregulate IL-6 expression in keratinocytes independent of p53 [23], suggesting that the baseline inflammatory milieu may differ from that of primary cells. To address this potential limitation, we included untreated IHGK cultures as negative controls in all assays and observed no increase in IL-8 expression or signaling pathway activation, indicating that baseline inflammatory signaling is low. We acknowledge that findings obtained with immortalized cells require validation in primary keratinocytes or organotypic models, but the consistency of responses across multiple studies and the availability of well-characterized IHGK lines support their use for mechanistic studies.

### 2.5. Real-Time Cell Analysis (xCELLigence)

The xCELLigence real-time cell analysis system (ACEA Biosciences, San Diego, CA, USA) was used to monitor impedance-based changes in gingival keratinocyte monolayers after exposure to composite dusts or eluates. Cell index (CI) values reflect a combined readout of cell number, cell spreading, cell–substrate contact, and monolayer integrity. Therefore, under the present experimental design, CI values were interpreted as post-exposure monolayer dynamics rather than as direct measurements of initial adhesion or unconstrained proliferation. For background measurement, 100 μL of KGM2 was added to each well of two 8-well E-Plates and placed in the RTCA analyzer at 37 °C and 5% CO_2_. Subsequently, 3 × 10^3^ HGKs were seeded per well in 500 μL KGM2. After 72 h, cells had formed a stable sub-confluent to near-confluent monolayer, and the medium was replaced with composite dust or eluate suspensions at 300 μg/mL. Real-time monitoring continued for at least 72 h after exposure. Because treatment was initiated after the initial attachment phase, this assay does not quantify initial adhesion. Instead, it assesses how dust and eluate exposure affect the stability, spreading, and impedance profile of an established keratinocyte layer.

### 2.6. ERK Pathway Inhibition (U0126)

To evaluate the involvement of ERK signaling in cellular responses triggered by composite materials, pharmacological inhibition of the ERK signaling pathway was performed using the MEK1/2 inhibitor U0126. U0126 (100 mM in DMSO; Merck, Darmstadt, Germany; Cat. No. 662009) was incubated with the HGKs at a final concentration of 15 μM in medium. The inhibitor was preincubated for 30 min before the addition of dust or eluates. The inhibitor was maintained throughout the subsequent treatment period. For the negative control, the HGKs were incubated with equal volumes of DMSO to exclude solvent-related effects.

### 2.7. Quantitative Real-Time PCR Analysis

Total RNA was extracted from treated HGKs using the RNeasy Mini Kit (Qiagen, Hilden, Germany) according to the manufacturer’s instructions. RNA concentration was measured using the QiaExpert system (Qiagen), and RNA integrity was assessed with the 4150 TapeStation system (Agilent, Santa Clara, CA, USA). One microgram of total RNA was reverse-transcribed into cDNA using the RevertAid First Strand cDNA Synthesis Kit (Thermo Fisher Scientific, Waltham, MA, USA) on a C1000 thermal cycler (Bio-Rad, Munich, Germany). Quantitative real-time PCR was performed using the CFX96 Touch Real-Time PCR Detection System (Bio-Rad) with RT^2^ SYBR Green PCR Master Mix (Qiagen). Each reaction contained 10 ng cDNA in a total volume of 25 μL. Cycling conditions were 95 °C for 10 min, followed by 40 cycles of 95 °C for 15 s and 60 °C for 1 min. Relative gene expression was calculated using the ΔΔCt method. UBC and RPL13A were used as reference genes. All RT^2^ qPCR primers (FLG, CXCL8, VIM, FN1, KRT1, KRT10, IVL, RELA, and SIRT1) were obtained from Qiagen (Hilden, Germany).

### 2.8. Indirect Immunofluorescence (IIF)

IIF staining was performed to assess the expression and subcellular distribution of fibronectin (FN1, Abcam, ab2413, Cambridge, UK), keratinocyte differentiation markers (KRT1, Abcam, ab185628 and KRT10, Abcam, ab76318), phospho-SIRT1 (Ser682; Creative Diagnostics, CABT-B1714, Shirley, NY, USA), and NF-κB phospho p65 (Ser536; Cell Signaling Technology, #3033, Danvers, MA, USA), as well as cytoskeletal organization. HGK cells were seeded on sterile 13 mm glass coverslips in 12-well plates and cultured for 72 h. Cells were then exposed to composite-derived dust or eluates (300 μg/mL), with KGM2 used as the negative control. After 24 h treatment, cells were washed with DPBS, fixed with 4% paraformaldehyde for 20 min, permeabilized with 0.1% Triton X-100 for 10 min, and blocked with 5% bovine serum albumin (BSA) for 30 min. Cells were incubated overnight at 4 °C with primary antibodies diluted in 1% BSA, followed by incubation with Alexa Fluor 488–conjugated secondary antibodies (Santa Cruz Biotechnology, Santa Cruz, CA, USA) for 1 h at room temperature in the dark. The actin cytoskeleton was counterstained with Texas Red, and nuclei were labeled with DAPI (300 nM) for 15 min. After mounting, fluorescence images were acquired using a Keyence BZ-9000 fluorescence microscope under identical exposure settings for all experimental groups. For SIRT1 and NF-κB analyses, fluorescence intensities were quantified separately in nuclear and cytoplasmic regions using ImageJ software 1.54t, and nuclear-to-cytoplasmic (N/C) intensity ratios were calculated.

### 2.9. Western Blot

Western blot analysis was performed to evaluate protein expression and phosphorylation status following composite dust or eluate exposure. After treatment, HGKs were washed with DPBS and lysed on ice in RIPA buffer (Sigma-Aldrich, Taufkirchen, Germany, #R0278L) supplemented with protease and phosphatase inhibitors (PhosSTOP™, Sigma-Aldrich, #04906845001). Cell lysates were clarified by centrifugation, and protein concentrations were determined using a BCA assay (Thermo Scientific, #23227). Equal amounts of protein were separated by a 4-15% Criterion™ TGX Stain-free™ Protein Gel (Bio-Rad, #5678084), transferred onto PVDF membranes (Bio-Rad, #1704275) and processed with the Bio-Rad turbo station and transferred with the pre-programmed Midi-gel protocol. Membranes were blocked with bovine serum albumin (BSA) (Thermo Scientific, #23208) and incubated overnight at 4 °C with primary antibodies against fibronectin (FN1; Abcam, ab2413) and the keratinocyte differentiation markers KRT1 (Abcam, ab185628) and KRT10 (Abcam, ab76318). SIRT1 protein levels were detected using antibodies against total SIRT1 (Abcam, ab110304) and phospho-SIRT1 (Ser682; Creative Diagnostics, CABT-B1714). Signaling pathway components were analyzed using antibodies against NF-κB p65 (Abcam, ab16502) and phospho-NF-κB p65 (Ser536; Cell Signaling Technology, #3033), ERK1/2 (Abcam, ab17942) and phospho-ERK1/2 (Thr202/Tyr204; Santa Cruz Biotechnology, sc-136521), as well as p38 MAPK (Abcam, ab170099) and phospho-p38 MAPK (Thr180/Tyr182; Cell Signaling Technology, #4511). After incubation with HRP-conjugated secondary antibodies, immunoreactive bands were visualized using enhanced chemiluminescence and imaged with a ChemiDoc™ Touch imaging system (Bio-Rad). For densitometric analyses, only exposure images within the linear detection range were used. Saturation was assessed during image acquisition using the ChemiDoc/Image Lab exposure series and saturated-pixel display. Bands containing saturated or clipped pixels were excluded from densitometric analysis. Where necessary, shorter exposure images from the same membrane were used for quantification. Representative Western blot images for publication were recorded or exported using the ChemiDoc extended dynamic range acquisition mode to visualize both low- and high-abundance signals across treatment groups. These representative display images were used for visualization only; quantitative densitometry was performed from the corresponding non-saturated exposure images. Band intensities were quantified using ImageJ software. Phosphorylation levels of ERK1/2, p38, and NF-κB were calculated as ratios of phosphorylated to total protein. β-Tubulin (Abcam, ab6046) was used as a loading control for normalization.

### 2.10. ELISA

IL-8/CXCL8 secretion was quantified using a commercially available human IL-8 ELISA kit (Quantikine^®^, R&D Systems, Minneapolis, MN, USA) according to the manufacturer’s instructions. After composite dust or eluate exposure, cell culture supernatants were collected and centrifuged to remove cellular debris. All samples were subsequently concentrated using centrifugal filter units with an appropriate molecular weight cutoff prior to analysis to ensure concentrations within the detection range of the assays. Absorbance was measured using a microplate reader, and IL-8 concentrations were calculated based on standard curves generated for each assay. Cytokine levels were normalized to total cellular protein content to account for differences in cell number.

### 2.11. Statistical Analysis

All experiments were performed with at least three independent biological replicates. Data are presented as mean ± standard deviation (SD). Statistical analyses were conducted using GraphPad Prism 9.0 (GraphPad Software, San Diego, CA, USA). Prior to parametric testing, data were assessed for normality and homogeneity of variances. For comparisons involving more than two groups, one-way analysis of variance (ANOVA) was applied. When the ANOVA indicated a statistically significant main effect, post hoc multiple comparisons were performed using Tukey’s honestly significant difference (HSD) test for all pairwise comparisons or Dunnett’s test when comparing each treatment to a single control. In analyses where only a small number of pre-specified comparisons were of interest, Bonferroni-adjusted Fisher’s LSD tests were used. These approaches control the family-wise type I error rate and are more conservative than an unadjusted LSD. Re-analysis of our data using Tukey’s or Bonferroni-adjusted comparisons did not materially alter the patterns of statistical significance described in the Results. A value of *p* < 0.05 (after adjustment for multiple comparisons) was considered statistically significant.

Sample size determination: Our experiments were performed with at least three independent biological replicates per condition. Because effect sizes for gingival keratinocyte responses to composite dust and eluates were unknown at the study’s outset, we did not perform a formal a priori power analysis. Instead, the sample size was guided by precedent from similar studies on composite dust effects [18] and by logistical considerations for cell-culture experiments. After data collection, we conducted post hoc power estimates using the observed variances and effect sizes. These calculations indicated that three independent replicates afforded >80% power to detect the observed differences at a significance level of 0.05, which is consistent with the conventional target power of 0.8 [24]. Nevertheless, we acknowledge that small sample sizes can reduce statistical power and increase the risk of Type II errors [24].

## 3. Results

### 3.1. Morphometric and Ultrastructural Characterization of Composite-Derived Particles

A dust particle analysis was conducted to compare morphologies among composites, given concerns that particle size and shape might influence keratinocyte responses. High-resolution SEM imaging of dust-adhered keratinocyte surfaces provided quantitative morphometric data for three restorative materials. Particles adhering to cells were segmented and their projected area, equivalent circular diameter, circularity, solidity and aspect ratio were calculated; particle density was expressed as number per mm^2^ of keratinocyte surface. Data from multiple fields per material (total ≈ 5000 particles) were pooled, and differences were tested with one-way ANOVA and Tukey’s post hoc comparisons. All composites generated predominantly micron-scale particles on cell surfaces, but their dimensions and shapes differed significantly by material.

**Figure 1:**
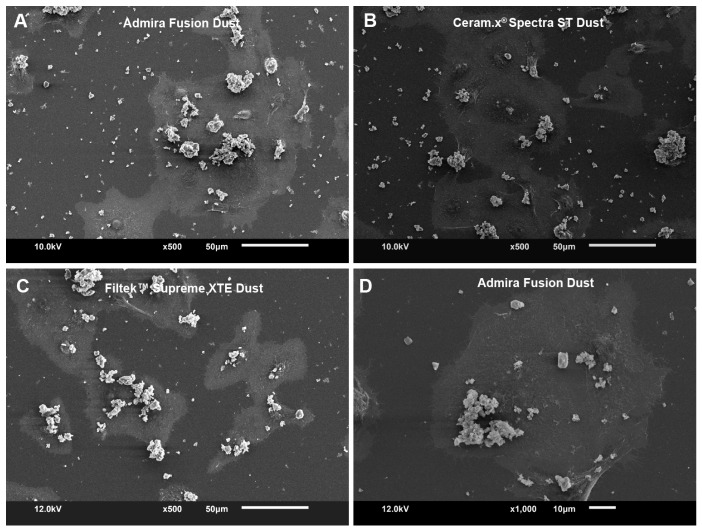
Scanning electron microscopy of composite dust on keratinocytes. (**A**–**C**) Representative SEM micrographs (500×) of human gingival keratinocyte monolayers exposed to polishing dust from three restorative composites. (**A**) Cells exposed to Admira Fusion ormocer dust are covered by relatively large, irregular fragments with lower particle density; (**B**) Ceram.x^®^ Spectra ST dust yields a dense coating of medium-sized clusters; (**C**) Filtek™ Supreme XTE nanofilled dust produces numerous small spherical aggregates and occasional larger agglomerates. (**D**) Higher magnification of Admira Fusion dust particles for exemplified particle documentation. Images were acquired at 10 kV accelerating voltage (**A**,**B**) or 12 kV (**C**); each composite was tested in duplicate independent experiments, and similar morphologies were observed in the second replicate. Scale bars, 50 μm and 10 μm in (**D**).

Admira Fusion dust fragments were largest, with a mean area of 1.6 ± 3.5 μm^2^ and an equivalent circular diameter of 1.4 ± 1.5 μm (Figure 1A, Table 1) by Ceram.x^®^ Spectra ST particles (1.0 ± 2.0 μm^2^, 1.1 ± 1.0 μm) and Filtek™ Supreme XTE particles, which were smallest (0.4 ± 1.5 μm^2^, 0.7 ± 0.8 μm); all size differences were significant (*p* < 0.05) (Figure 1B,C, Table 1).

Circularity increased in the order Admira Fusion (0.72 ± 0.15) < Ceram.x^®^ Spectra ST (0.80 ± 0.10) < Filtek™ Supreme XTE (0.88 ± 0.07), while solidity followed the same trend (0.92 ± 0.08 < 0.96 ± 0.04 < 0.98 ± 0.02); all pairwise differences were significant (*p* < 0.05). Aspect ratio decreased from 1.8 ± 0.5 (Admira Fusion) to 1.4 ± 0.3 (Ceram.x^®^ Spectra ST) to 1.2 ± 0.2 (Filtek™ Supreme XTE), again with significant differences (*p* < 0.05). Thus, Filtek™ Supreme XTE dust produced the most spherical and compact particles, Admira Fusion the most irregular and elongated, and Ceram.x^®^ Spectra ST particles were intermediate (Figure 1A–C).

The number of particles adhering per mm^2^ of keratinocyte surface was highest for Filtek™ Supreme XTE (1.5 × 10^5^ ± 2 × 10^4^ mm^−2^), intermediate for Ceram.x^®^ Spectra ST (1.0 × 10^5^ ± 1 × 10^4^ mm^−2^), and lowest for Admira Fusion (5.0 × 10^4^ ± 5 × 10^3^ mm^−2^); each difference was significant (*p* < 0.05).

In summary, Filtek™ Supreme XTE dust yields numerous small, nearly spherical particles that densely blanket keratinocyte surfaces; Admira Fusion dust produces fewer, larger and more irregular particles (Figure 1D); and Ceram.x^®^ Spectra ST dust displays intermediate size and shape. These quantitative differences provide a basis for correlating material composition with cellular responses.

Because the composites generated particles with markedly different dimensions and densities, we performed an exploratory normalization using SEM-derived projected particle area and particle density. The estimated projected particle contact burden was calculated as mean particle area × particle density. This yielded approximately 8.0 × 10^4^ μm^2^/mm^2^ for Admira Fusion, 1.0 × 10^5^ μm^2^/mm^2^ for Ceram.x^®^ Spectra ST, and 6.0 × 10^4^ μm^2^/mm^2^ for Filtek™ Supreme XTE. Thus, the inflammatory response observed for Admira Fusion was not simply proportional to estimated projected surface coverage, because Ceram.x^®^ Spectra ST displayed the highest estimated coverage but did not induce IL-8 secretion. However, this calculation is based on projected two-dimensional SEM area and does not account for true three-dimensional surface area, material density, sedimentation behavior, surface charge, or nanoscale surface chemistry. Therefore, particle burden and sedimentation remain important limitations when interpreting material-specific biological effects.

Because aggregate-rich particle morphologies may influence epithelial adhesion, proliferation, and downstream stress-responsive signaling, we examined in a further step whether these material-specific morphometric properties correspond to differential effects on keratinocyte behavior.

### 3.2. Composite Dust Alters Impedance-Based Keratinocyte Monolayer Dynamics More Strongly than Eluates

Given the critical role of keratinocyte proliferation in maintaining gingival epithelial integrity, we assessed whether composite-derived dusts and eluates differentially affected this function due to their distinct physical and chemical properties.

**Figure 2:**
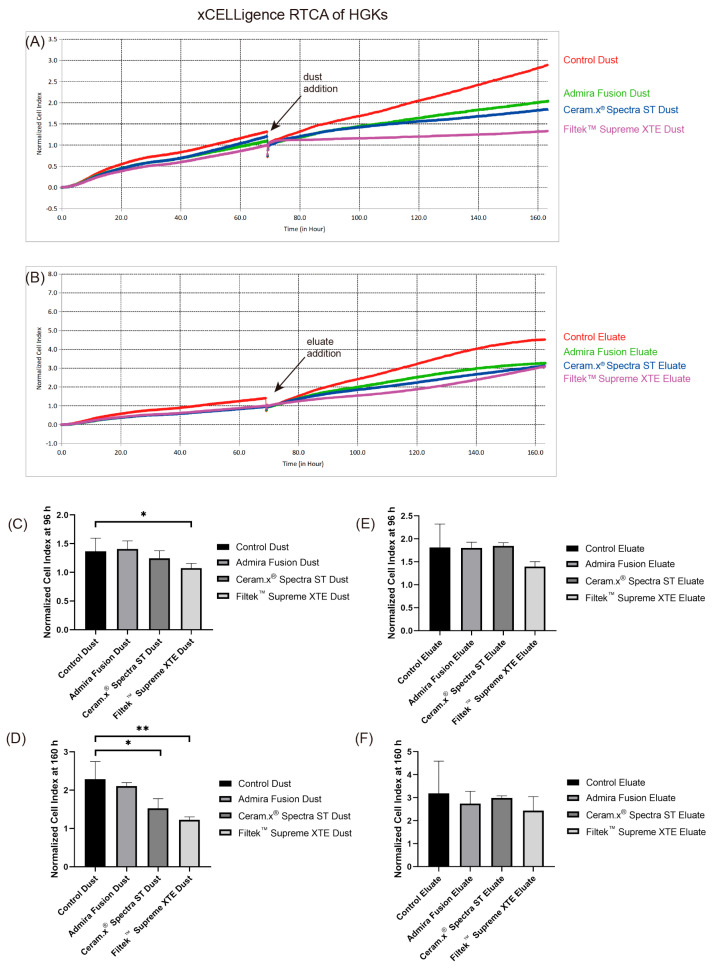
Composite dust alters impedance-based keratinocyte monolayer dynamics more strongly than corresponding eluates. (**A**,**B**) Real-time impedance-based monitoring (xCELLigence RTCA) of human gingival keratinocytes (HGKs; HPV16 E6/E7–immortalized) cultured for 72 h and then exposed to composite-derived dust (**A**) or the corresponding eluates (**B**) at 300 μg/mL. Arrows indicate the time of dust/eluate addition (medium exchange). Traces show normalized cell index (nCI) recorded up to 160 h. Because treatments were applied after 72 h of culture, CI changes reflect post-exposure monolayer dynamics, including cell spreading, cell–substrate contact, and monolayer integrity, rather than initial adhesion. (**C**,**D**) Quantification of nCI at 96 h (**C**) and 160 h (**D**) for dust-treated cells. (**E**,**F**) Quantification of nCI at 96 h (**E**) and 160 h (**F**) for eluate-treated cells. Bars represent mean ± SD from independent experiments (*n* ≥ 3). Statistical significance was assessed using one-way ANOVA with Dunnett’s post hoc test. * *p* < 0.05; ** *p* < 0.01. CI, cell index; nCI, normalized cell index.

Real-time impedance monitoring using xCELLigence revealed pronounced differences in cell index (CI) dynamics between dust- and eluate-treated HGKs (Figure 2A,B). Dusts from Filtek™ Supreme XTE and Ceram.x^®^ Spectra ST induced rapid and sustained reductions in CI of approximately 25% and 10% at 96 h, respectively, compared to untreated controls (*p* < 0.05; Figure 2C). These inhibitory effects became more pronounced over time and persisted throughout the 160h observation period, with CI reductions reaching approximately 45% for Filtek™ Supreme XTE and 35% for Ceram.x^®^ Spectra ST at 160 h (*p* < 0.05–0.01; Figure 2D). The pronounced divergence of the CI curves at later time points (e.g., by 160 h) suggests that dust-treated keratinocytes also suffer a sustained proliferation deficit compared to control cells.

In contrast, Admira Fusion dust caused only a mild and transient CI reduction of approximately 5–10% at 96 h, which remained limited at later time points.

By comparison, eluates derived from the same materials produced only modest changes in CI (Figure 2B). At 96 h, Filtek™ Supreme XTE eluate induced an approximate 20% reduction in CI, whereas Ceram.x^®^ Spectra ST and Admira Fusion eluates showed minimal or no inhibitory effects relative to control conditions (Figure 2E). These trends were largely maintained at 160 h, with Filtek™ Supreme XTE eluate causing a mild CI reduction of approximately 15–20%, while Ceram.x^®^ Spectra ST eluate displayed CI values comparable to or slightly higher than control, and Admira Fusion eluate showed no significant impact over the entire measurement period (*p* > 0.05; Figure 2F).

Together, these findings demonstrate that composite-derived dusts exert stronger inhibitory effects on keratinocyte proliferation and adhesion than their corresponding eluates, with material-specific variability in the extent of impairment.

To determine whether these functional impairments may indicate broader disturbances in epithelial maturation, we next examined whether composite exposure also affects keratinocyte differentiation markers.

### 3.3. Composite Dusts and Eluates Downregulate Keratinocyte Differentiation Markers at Transcriptional and Protein Levels

To investigate whether composite-derived dusts and eluates compromise keratinocyte differentiation, a key cell function for gingival epithelial barrier maintenance, we quantified expression levels of hallmark epithelial markers, KRT1, KRT10, FLG, and IVL, at the transcriptional level and validated selected findings at the protein level.

**Figure 3:**
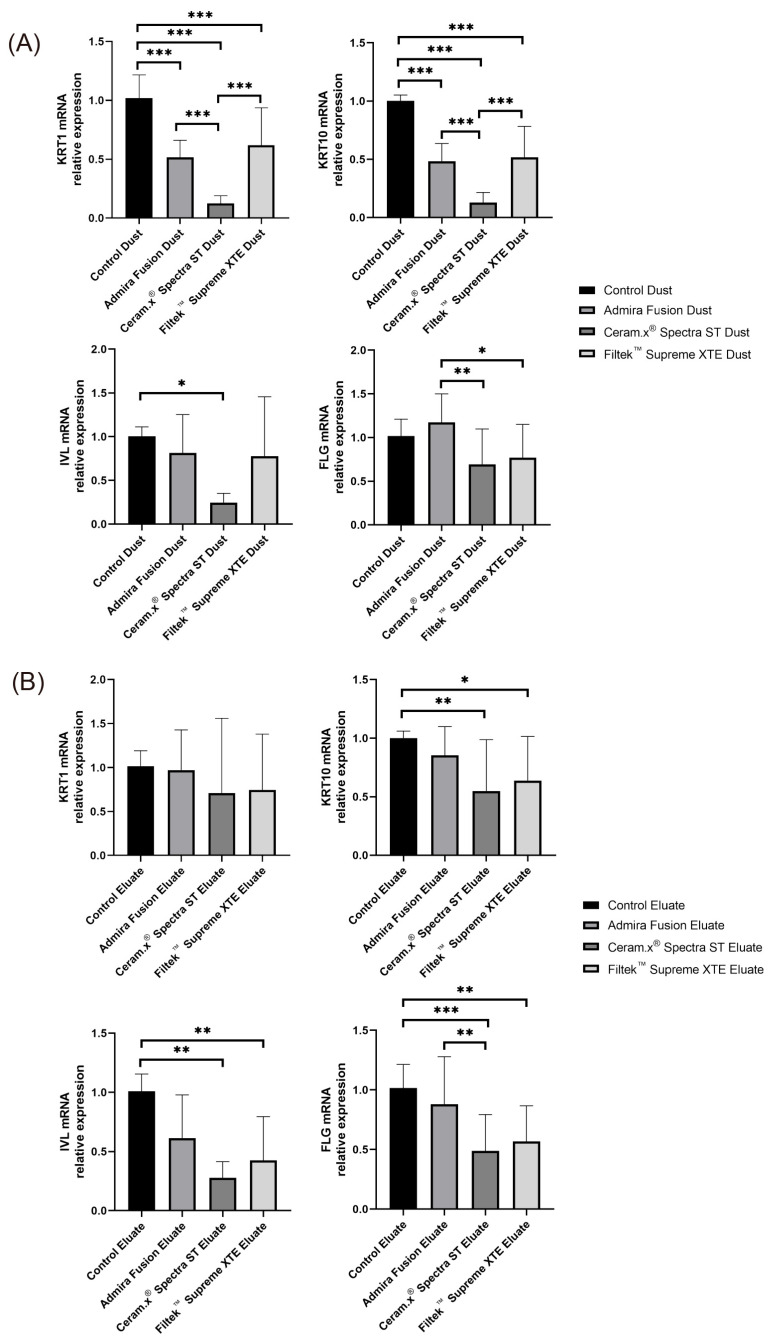
Dust and eluate exposure suppress gingival keratinocyte differentiation markers. (**A**) Relative mRNA expression of early KRT1, KRT10 and late IVL, FLG differentiation markers in gingival keratinocytes after 24 h exposure to composite dusts. Cells were treated with dusts generated from Admira Fusion, Ceram.x^®^ Spectra ST, or Filtek™ Supreme XTE, and transcripts were quantified by qRT-PCR and normalized to β-actin. (**B**) Relative expression of the same markers following exposure to eluates from the corresponding composites for 24 h. Bars represent means ± SD of three independent experiments normalized to the control (dust or eluate, respectively). Differences between groups were assessed by one-way ANOVA followed by Dunnett’s post hoc comparison to the control condition; * *p* < 0.05; ** *p* < 0.01; *** *p* < 0.001.

Exposure for 24 h to dust from Filtek™ Supreme XTE and Ceram.x^®^ Spectra ST led to significant downregulation of KRT1 and KRT10 mRNA levels, with reductions ranging approximately from 40% to 90% relative to untreated controls (*p* < 0.001; Figure 3A). IVL transcripts were similarly decreased (approximately 70–80%), while FLG expression showed a more moderate reduction (~30–40%). Eluates from these materials induced generally smaller but still detectable decreases in differentiation marker expression. In contrast, Admira Fusion dust and eluate produced comparatively minor changes in mRNA levels, with most differences not reaching statistical significance (*p* > 0.05).

**Figure 4:**
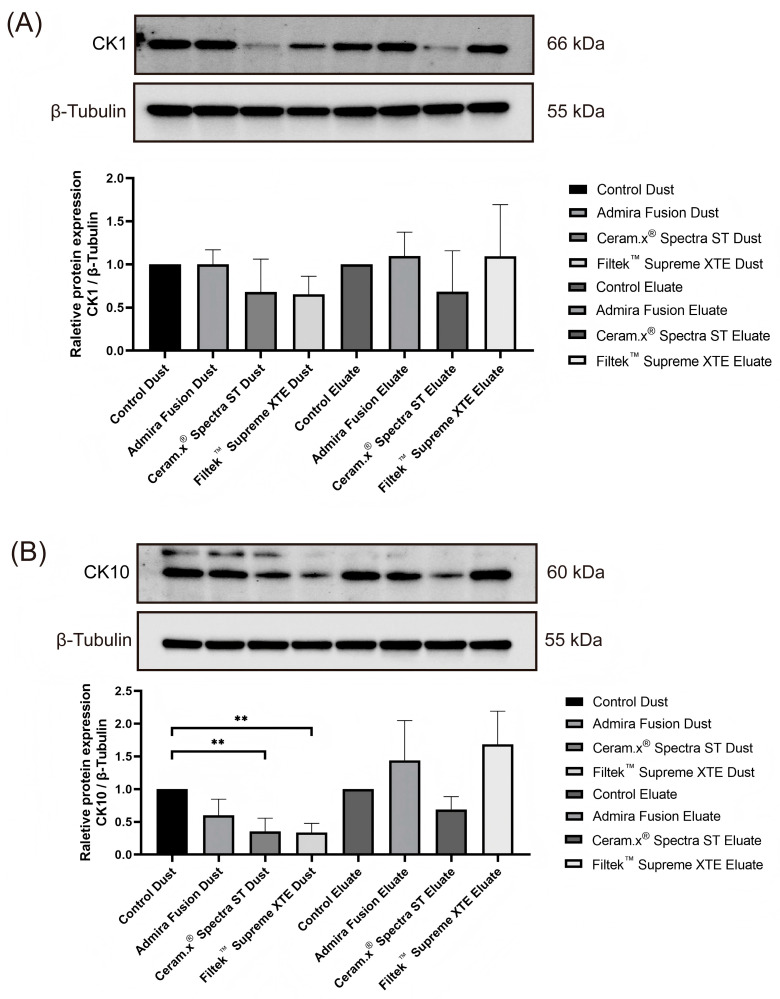
Keratin 1 and keratin 10 protein levels after exposure to composite dusts and eluates. (**A**) Representative immunoblots showing CK1 (~66 kDa) in gingival keratinocytes exposed for 24 h to dusts or eluates from Admira Fusion, Ceram.x^®^ Spectra ST or Filtek™ Supreme XTE. β-Tubulin (~55 kDa) was used as a loading control. Densitometric quantification (lower panel) shows CK1 levels normalized to β-tubulin relative to the corresponding control. (**B**) Representative blots and quantification for CK10 (~60 kDa) with β-tubulin loading control. Bar graphs represent mean ± SD from three independent experiments for each condition (Control Dust, Admira Fusion Dust, Ceram.x^®^ Spectra ST Dust, Filtek™ Supreme XTE Dust, Control Eluate, Admira Eluate, CeramX Eluate, Filtek™ Supreme XTE Eluate). Statistical differences compared with the control dust condition were assessed by one-way ANOVA and Dunnett’s post hoc test; ** *p* < 0.01. Densitometric quantification was performed using non-saturated exposure images within the linear detection range.

To assess whether these transcriptomic changes translated to the protein level, we performed Western blot analysis for KRT1 and KRT10, which represent early markers of keratinocyte differentiation and are indicative of the initiation of epithelial maturation. We focused on these markers because IVL and FLG are predominantly expressed during later stages of differentiation, and early markers provide a robust readout of differentiation status within the 24 h composite exposure window. WB analysis confirmed marked reductions in KRT1 and KRT10 protein levels in dust-exposed keratinocytes (Figure 4A,B). Quantification revealed decreases of approximately 45–50% for Filtek™ Supreme XTE and ~40–70% for Ceram.x^®^ Spectra ST, depending on the marker analyzed (*p* < 0.001; Figure 4A,B), whereas their eluates caused milder reductions of approximately 21–26% (*p* < 0.05). Admira Fusion dust and eluate did not significantly alter protein expression compared to controls (*p* > 0.05).

Together, these findings indicate that composite-derived dusts, particularly from Filtek™ Supreme XTE and Ceram.x^®^ Spectra ST, more potently downregulate keratinocyte differentiation markers than their eluates, suggesting material-specific and dust- versus eluate-dependent impairments of this critical cell function at both transcriptional and early protein-expression levels.

Because keratinocyte differentiation is tightly linked to extracellular matrix assembly, we next examined whether these alterations extend to fibronectin expression as a key ECM component.

### 3.4. Particulate Versus Eluate Exposure Drives Distinct Fibronectin Expression Patterns in Gingival Keratinocytes

Given the essential role of FN1 in maintaining gingival epithelial architecture, cell adhesion, and wound healing, we hypothesized that composite-derived dusts and eluates would differentially modulate FN1 expression, reflecting material-specific and exposure-dependent ECM alterations. To address this, we quantified FN1 mRNA levels in HGKs after 24 h exposure to composite dusts and eluates by RT-qPCR, and assessed protein expression by WB analysis.

**Figure 5:**
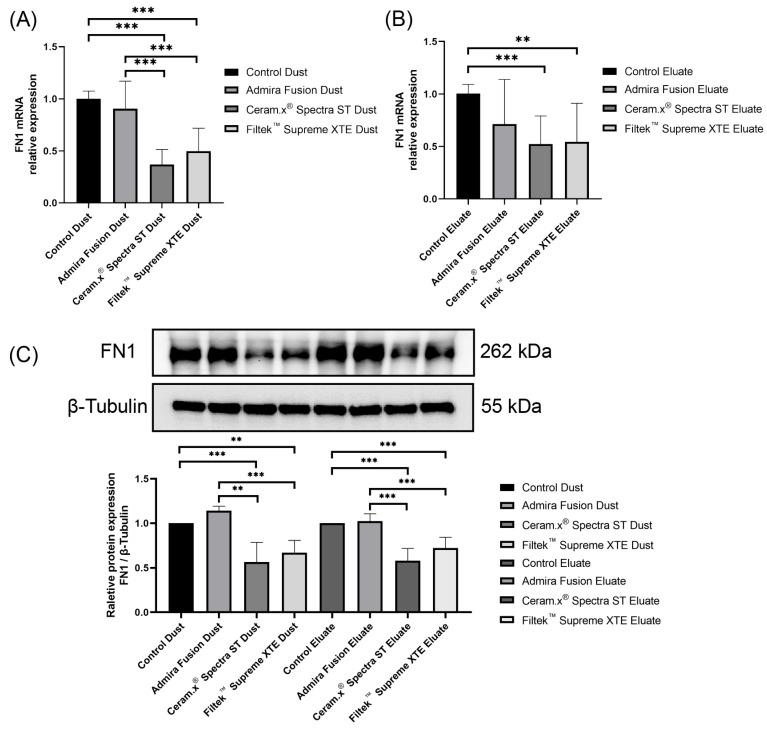
Dust and eluate exposure reduce fibronectin expression in gingival keratinocytes. (**A**) Quantitative RT–PCR analysis of FN1 mRNA relative to β-actin in cells exposed for 24 h to dusts derived from Admira Fusion, Ceram.x^®^ Spectra ST or Filtek™ Supreme XTE. Values are normalized to the control dust condition. (**B**) Relative FN1 mRNA expression after exposure to composite eluates for 24 h. (**C**) Representative immunoblot of FN1 (~262 kDa) with β-tubulin (~55 kDa) as a loading control and densitometric quantification of FN1/β-tubulin ratios. Bars represent mean ± SD from three independent experiments for each condition (Control Dust, Admira Fusion Dust, Ceram.x^®^ Spectra ST Dust, Filtek™ Supreme XTE Dust, Control Eluate, Admira Fusion Eluate, Ceram.x^®^ Spectra ST Eluate, Filtek™ Supreme XTE Eluate). Statistical significance was assessed using one-way ANOVA with Dunnett’s post hoc test; ** *p* < 0.01; *** *p* < 0.001.

Exposure to dust from Filtek™ Supreme XTE and Ceram.x^®^ Spectra ST significantly reduced FN1 transcript levels by approximately 50–65% relative to untreated controls (*p* < 0.001; Figure 5A). The corresponding eluates resulted in more moderate reductions (~45–50%; Figure 5B). In contrast, Admira Fusion dust largely preserved FN1 mRNA expression, exhibiting only a minor reduction (~10–15%) that did not reach statistical significance (*p* > 0.05), while its eluate showed a numerically lower FN1 level (~25–30% reduction) that likewise did not reach significance.

At the protein level, Western blot analysis largely corroborated the transcriptional trends. Dust from Filtek™ Supreme XTE and Ceram.x^®^ Spectra ST reduced FN1 protein levels by approximately 45–55% (*p* < 0.001; Figure 5C), whereas eluates produced milder decreases (~35–40%; *p* < 0.05). Admira Fusion dust comparatively preserved FN1 protein expression (*p* > 0.05), and its eluate similarly did not significantly alter FN1 levels.

**Figure 6:**
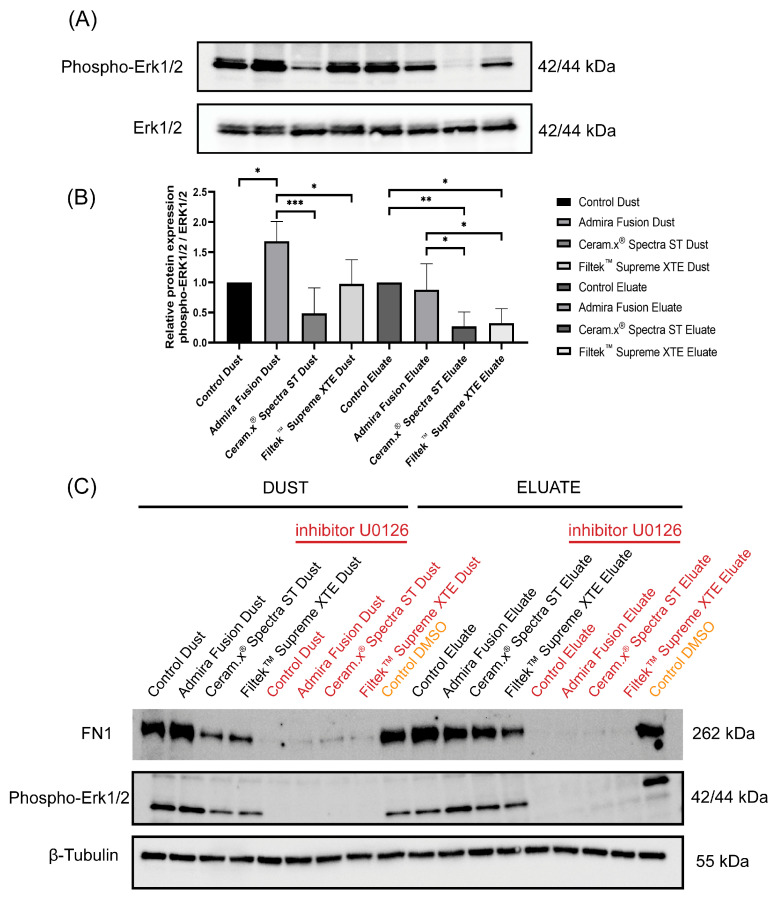
Composite dust and eluate exposures modulate ERK1/2 activation and fibronectin expression. (**A**) Representative immunoblots of phosphorylated ERK1/2 (p-Erk1/2) and total ERK1/2 in gingival keratinocytes after 24 h exposure to dusts or eluates derived from Admira Fusion, Ceram.x^®^ Spectra ST or Filtek™ Supreme XTE. Samples were normalized for equal protein loading. (**B**) Densitometric quantification of p-Erk1/2 relative to total Erk1/2 for the conditions shown in (**A**). Bars represent mean ± SD from three independent experiments; significance was determined by one-way ANOVA with appropriate post hoc testing (* *p* < 0.05, ** *p* < 0.01, *** *p* < 0.001). (**C**) Representative immunoblots for FN1 and p-Erk1/2 in keratinocytes exposed to composite dusts or eluates in the absence or presence of the MEK inhibitor U0126. Cells were pre-incubated with U0126 or vehicle (DMSO) for 1 h before treatment; β-tubulin served as a loading control. Specific inhibitor samples are indicated in red in the figure and the corresponding control in orange. Densitometric quantification was performed using non-saturated exposure images selected from the ChemiDoc/Image Lab exposure series. Representative blots were recorded or exported in extended dynamic range mode to visualize both weak and strong signals across treatment groups and were not used for quantification when saturation was present or suspected.

To probe potential mechanistic underpinnings, ERK1/2 inhibition with U0126 produced a comparable reduction in FN1 protein (~50%; *p* < 0.01; Figure 6C), suggesting that ERK signaling may contribute to FN1 regulation and that FN1 suppression under Filtek™ Supreme XTE and Ceram.x^®^ Spectra ST exposure coincides with reduced ERK activity.

Together, these results demonstrate that composite-derived dusts, particularly those from Filtek™ Supreme XTE and Ceram.x^®^ Spectra ST, more potently downregulate FN1 expression than their eluates, whereas Admira Fusion largely preserves FN1 levels. Given this ERK-associated regulation of FN1, we next investigated whether composite-derived dusts and eluates differentially affect ERK1/2 activation as a potential upstream mechanism.

### 3.5. Composite Dusts and Eluates Differentially Modulate ERK1/2 Signaling

Given the established role of ERK signaling in orchestrating keratinocyte proliferation, differentiation, and fibronectin deposition, we next hypothesized that composite-derived dusts and eluates might differentially perturb ERK1/2 activation, reflecting material- and exposure-dependent regulatory patterns. To test this, we analyzed phosphorylated ERK1/2 (pERK1/2) protein levels by Western blot following 24 h exposure of HGKs to composite dusts and eluates.

Dust exposure from Filtek™ Supreme XTE and Ceram.x^®^ Spectra ST composites strongly suppressed ERK1/2 phosphorylation, reducing pERK1/2 levels by roughly 60–70% compared to untreated controls (i.e., pERK1/2 was lowered to about 30–40% of baseline; Figure 6A,B). Their corresponding eluate exposures also inhibited ERK1/2 activation, but this effect was less pronounced—pERK1/2 levels remained at approximately 65–75% of control (a 25–35% decrease; *p* < 0.05). By contrast, Admira Fusion dust induced a modest yet significant increase in ERK1/2 phosphorylation (approximately 60–70% above control; *p* < 0.05), whereas its eluate had no significant effect on pERK1/2. Notably, total ERK1/2 protein levels were unchanged across all conditions, indicating that these differences reflect altered phosphorylation status rather than changes in overall ERK1/2 expression.

To test whether ERK activity contributes to FN1 regulation, the MEK1/2 inhibitor U0126 was applied, which effectively abrogated ERK1/2 phosphorylation and reduced FN1 protein expression by ~50% (*p* < 0.01; Figure 6C), supporting a role for ERK signaling in maintaining basal FN1 levels in HGKs. Although U0126-mediated ERK inhibition reduced FN1 expression by approximately 50%, we did not evaluate NF-κB p65 nuclear localization or p38 phosphorylation after U0126 treatment. Consequently, our data demonstrate that ERK contributes to FN1 regulation, but they do not allow us to determine whether ERK inhibition modulates downstream inflammatory pathways. Previous studies have shown that fibronectin fragments can activate TLR4-dependent p38/MK2 and NF-κB signaling, leading to IL-8 expression [13]. It is therefore plausible that FN1 acts downstream of ERK but upstream of p38/NF-κB; however, confirming this mechanistic sequence will require targeted perturbation of FN1 or these kinases in future work.

Together, these data identify ERK1/2 as a key regulatory pathway affected by composite exposure, with Filtek™ Supreme XTE and Ceram.x^®^ Spectra ST inducing a suppression-like phenotype characterized by ERK downregulation, and Admira Fusion dust eliciting an activation-like phenotype via ERK hyperactivation.

Given that ERK1/2 can modulate downstream inflammatory transcription factors and nuclear stress regulators, we next examined SIRT1 phosphorylation and NF-κB signaling to delineate material-dependent inflammatory and stress-response pathways.

### 3.6. SIRT1-S682 Profiles Under Particulate Exposure

**Figure 7:**
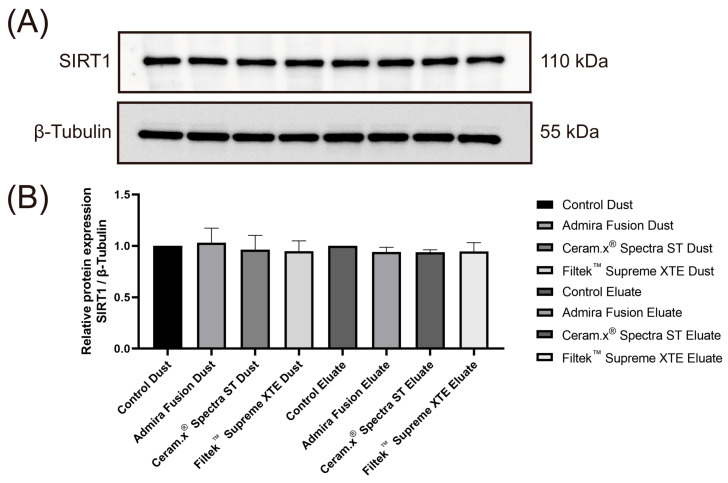
SIRT1 protein levels in gingival keratinocytes after exposure to composite dusts and eluates. (**A**) Representative immunoblots for SIRT1 (≈110 kDa) in cells exposed for 24 h to dusts or eluates generated from the composites Admira Fusion, Ceram.x^®^ Spectra ST and Filtek™ Supreme XTE. β-Tubulin served as a loading control. (**B**) Densitometric quantification of SIRT1 normalized to β-tubulin. Bars represent mean ± SD from three independent experiments for each condition (Control Dust, Admira Fusion Dust, Ceram.x^®^ Spectra ST Dust, Filtek™ Supreme XTE Dust, Control Eluate, Admira Fusion Eluate, Ceram.x^®^ Spectra ST Eluate, Filtek™ Supreme XTE Eluate). Statistical analysis by one-way ANOVA revealed no significant differences. Densitometric quantification was performed using non-saturated exposure images within the linear detection range.

To determine whether composite-derived particulate stimuli influence nuclear SIRT1 regulatory status in HGKs, we first assessed total SIRT1 protein abundance across all dust- and eluate-treated groups. Western blot analysis showed that total SIRT1 levels remained unchanged under all conditions (Figure 7A,B), indicating that composite exposure does not alter overall SIRT1 expression.

Because the strongest functional alterations were consistently observed under particulate exposure, including impaired proliferation, disrupted differentiation, and ERK–FN1 modulation, subsequent analyses focused on SIRT1 phosphorylation at Ser682 (S682) in dust-treated cells.

**Figure 8:**
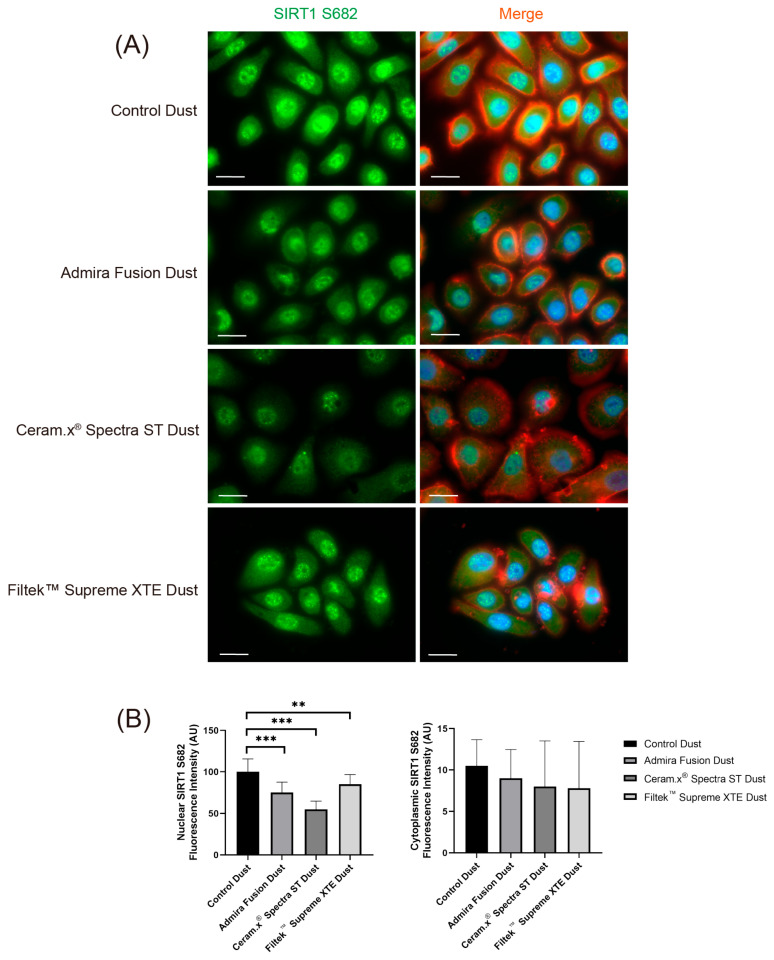
Localization and quantification of SIRT1 Ser682 phosphorylation following dust exposure. (**A**) Immunofluorescence staining of phospho-SIRT1 at Ser682 (green) in gingival keratinocytes exposed for 24 h to dusts generated from Admira Fusion, Ceram.x^®^ Spectra ST or Filtek™ Supreme XTE. Nuclei were counterstained with DAPI (blue) and F-actin with phalloidin (red); representative merged images (right) show subcellular distribution. Scale bars, 20 μm. The original images of the merged ones are in the Supplementary Materials. (**B**) Quantification of nuclear (left) and cytoplasmic (right) phospho-SIRT1 Ser682 fluorescence intensity. Values represent mean ± SD from at least 30 cells per condition in three independent experiments. Statistical differences were assessed using one-way ANOVA with Dunnett’s post hoc test; ** *p* < 0.01; *** *p* < 0.001.

Immunofluorescence revealed a dust-associated reduction in SIRT1-S682 abundance across all particulate exposure conditions. Admira Fusion dust and Ceram.x^®^ Spectra ST dust both reduced nuclear SIRT1-S682 relative to untreated controls, whereas Filtek™ Supreme XTE dust elicited the strongest decrease (Figure 8A, Beyond ECM control, B). Cytoplasmic SIRT1-S682 showed a similar downward trend across materials, while total SIRT1 protein abundance remained unchanged (Figure 7A,B). Thus, particulate exposure altered the phosphorylated SIRT1-S682 pool without detectably changing total SIRT1 expression. Because we did not measure SIRT1 deacetylase activity or manipulate SIRT1 phosphorylation, these data should be interpreted as a correlative marker of stress-associated nuclear regulation rather than as direct evidence of altered SIRT1 enzymatic function. Importantly, the reduction in SIRT1-S682 occurred across all particulate exposures, whereas IL-8 secretion was induced only by Admira Fusion dust. Therefore, SIRT1-S682 reduction alone was not sufficient to explain the inflammatory phenotype. We next assessed whether this shared SIRT1-S682 response was accompanied by material-dependent differences in NF-κB p65 abundance, localization, and activation state.

### 3.7. NF-κB p65 Abundance, Nuclear Localization, and Activation State Under Particulate Exposure

**Figure 9:**
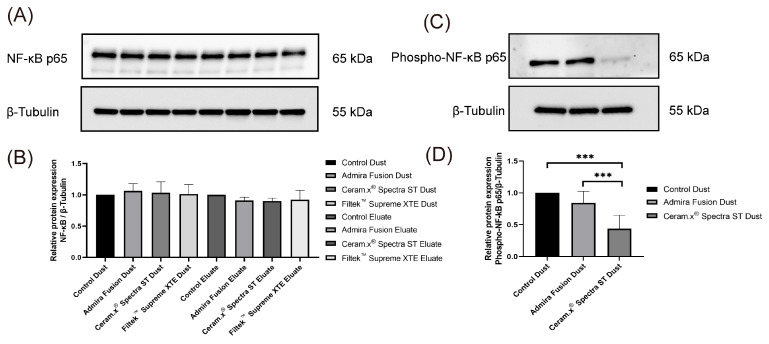
Composite dust and eluate exposures influence NF-κB p65 activation in gingival keratinocytes. (**A**) Representative immunoblots of total NF-κB p65 in cells exposed for 24 h to dusts or eluates generated from Admira Fusion, Ceram.x^®^ Spectra ST, or Filtek™ Supreme XTE. β-Tubulin served as the loading control. (**B**) Densitometric analysis of NF-κB p65 normalized to β-tubulin for the conditions in (**A**). Bars represent mean ± SD from three independent experiments; no significant differences were detected. (**C**) Immunoblot of phosphorylated NF-κB p65 (phospho-Ser536) in keratinocytes exposed to dusts from the indicated composites for 24 h, with β-tubulin as loading control. (**D**) Quantification of phospho-NF-κB p65 normalized to β-tubulin. Bars indicate mean ± SD from three independent experiments. Statistical differences were determined by one-way ANOVA with Dunnett’s post hoc test; *** *p* < 0.001. Densitometric quantification was performed using non-saturated exposure images within the linear detection range.

To evaluate whether composite-derived particulate stimuli influence NF-κB regulation in HGKs, we first quantified total p65 abundance across all dust-treated groups. Western blot analysis did not reveal a statistically significant or consistent material-dependent change in total NF-κB p65 protein abundance (Figure 9A,B). Although total p65 quantification showed biological variability across independent experiments, no reproducible directional trend was observed among the treatment groups.

**Figure 10:**
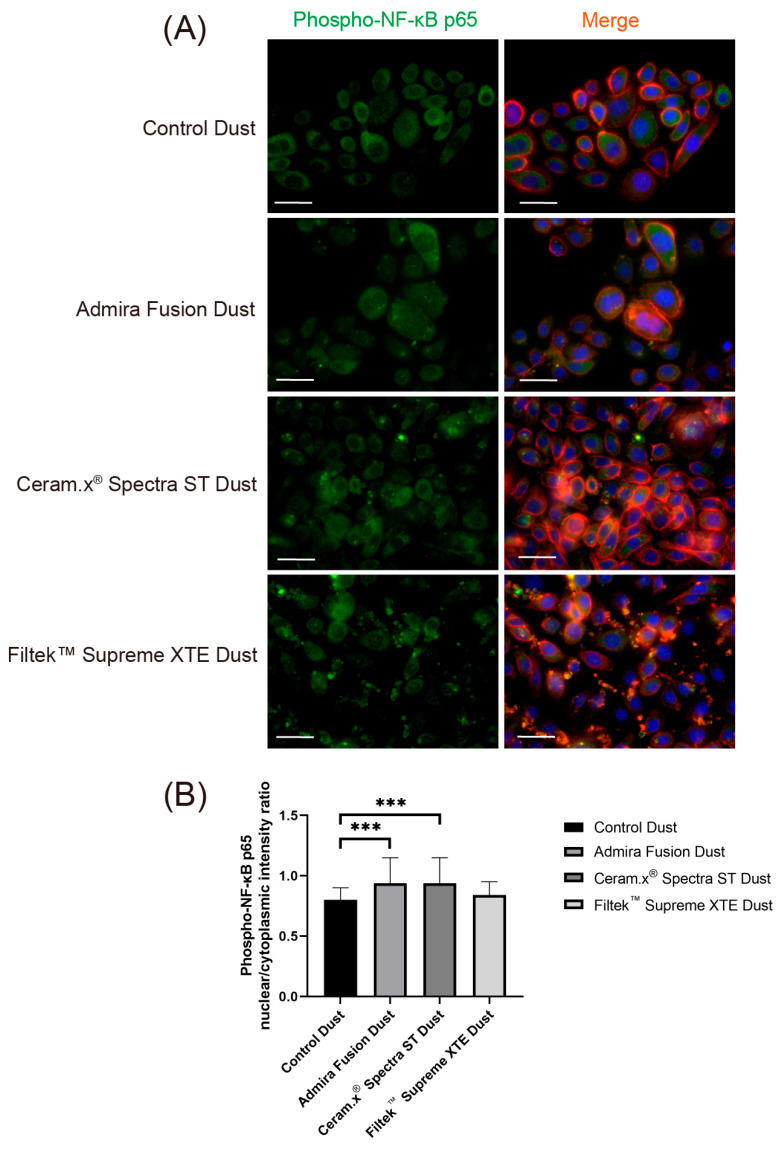
Localization and quantification of phosphorylated NF-κB p65 in gingival keratinocytes following composite dust exposure. (**A**) Representative confocal images showing phosphorylated NF-κB p65 (green) in keratinocytes exposed for 24 h to dusts generated from the indicated composites. Merged images also display F-actin (red) and DAPI-labeled nuclei (blue). Scale bars, 40 μm. The original images of the merged ones are in the Supplementary Materials. (**B**) Quantification of the nuclear-to-cytoplasmic fluorescence intensity ratio for phospho-NF-κB p65 in cells from three independent experiments (mean ± SD). Statistical significance between control dust and the indicated composite dust conditions was determined using one-way ANOVA with appropriate post hoc testing; *** *p* < 0.001.

Therefore, total p65 abundance was interpreted as a baseline protein-expression readout rather than as evidence for material-specific NF-κB activation. Because unchanged total p65 levels do not exclude differences in NF-κB signaling, we next analyzed subcellular p65 distribution and Ser536 phosphorylation as activation-related readouts. Quantification of the nuclear-to-cytoplasmic (N/C) fluorescence intensity ratio showed a clear material-dependent hierarchy (Figure 10A,B): Admira Fusion dust and Ceram.x^®^ Spectra ST dust both induced a marked increase in NF-κB nuclear accumulation (N/C ≈ 0.94), whereas Filtek™ Supreme XTE dust caused only a modest elevation (N/C ≈ 0.84) relative to untreated controls (N/C ≈ 0.80). Thus, Filtek™ Supreme XTE was not omitted from the NF-κB analysis; rather, it displayed a near-baseline localization profile under the present conditions.

Based on this quantitative localization pattern, subsequent phosphorylation-focused analyses were concentrated on Admira Fusion and Ceram.x^®^ Spectra ST, the two conditions showing clearer nuclear p65 enrichment. This focused analysis should not be interpreted as proof that Filtek™ Supreme XTE lacks any NF-κB pathway modulation. Instead, the data indicate that Filtek™ Supreme XTE induced only minimal nuclear p65 enrichment and did not increase IL-8 secretion above baseline under the present high-dose in vitro conditions. A complete phosphorylation analysis of all materials would be required to detect potential low-amplitude NF-κB activation events.To determine whether the nuclear p65 pool generated by Admira Fusion and Ceram.x^®^ Spectra ST dust was transcriptionally competent, we examined Ser536 phosphorylation, a hallmark of NF-κB activation (Figure 9C). In this focused comparison, Admira Fusion dust showed comparatively preserved p65-Ser536 phosphorylation, whereas Ceram.x^®^ Spectra ST dust showed a stronger decrease, consistent with a predominantly transcriptionally inactive NF-κB pool.

Both Admira Fusion and Ceram.x^®^ Spectra ST dust increased nuclear NF-κB accumulation, yet particulate exposure revealed clearly divergent NF-κB activation profiles. Although both materials reduced p65-Ser536 relative to control, dephosphorylation was substantially more pronounced under Ceram.x^®^ Spectra ST, yielding a predominantly Ser536-dephosphorylated and transcriptionally inactive NF-κB pool (Figure 9C and Figure 10A,B). In contrast, Ser536 levels remained comparatively preserved under Admira Fusion, consistent with the presence of a more transcriptionally competent NF-κB state. This divergence between nuclear presence and activation competence underscores the material-dependent regulation of NF-κB under particulate exposure.

Because both SIRT1 and NF-κB displayed material-specific alterations, we next examined p38-MAPK as an upstream stress-responsive kinase that could account for the observed differences in NF-κB phosphorylation states.

### 3.8. p38-MAPK Phosphorylation Following Dust Exposure

**Figure 11:**
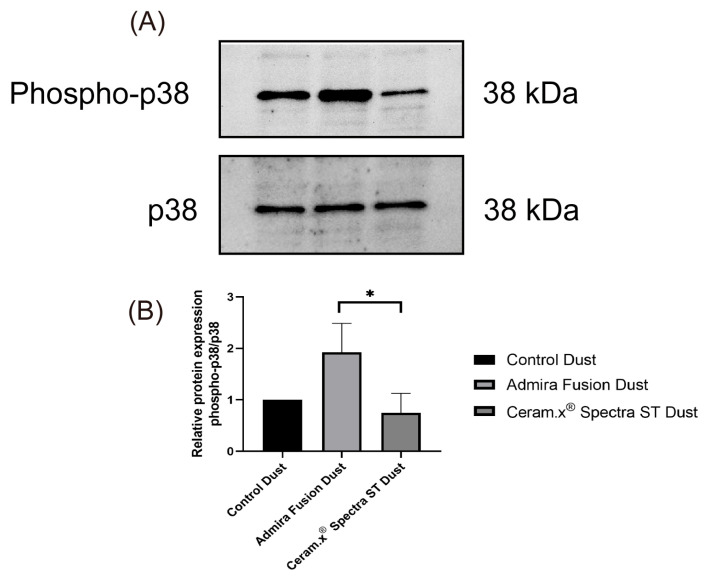
Effect of composite dusts on p38 MAPK phosphorylation. (**A**) Representative immunoblots of phosphorylated p38 (phospho-p38, ~38 kDa) and total p38 (~38 kDa) in gingival keratinocytes exposed for 24 h to control dust, Admira Fusion dust, or Ceram.x^®^ Spectra ST dust. (**B**) Densitometric quantification of phospho-p38 relative to total p38 for the conditions in (**A**). Bars represent mean ± SD from three independent experiments; statistical significance was determined by one-way ANOVA with appropriate post hoc testing; * *p* < 0.05. Densitometric quantification was performed using non-saturated exposure images within the linear detection range.

To determine whether composite-derived particulate stimuli engage stress-responsive MAPK pathways potentially linked to NF-κB regulation, we quantified phosphorylated p38 (phospho-p38) relative to total p38 under particulate exposure conditions. Normalization of phospho-p38 to total p38 revealed a clear, material-specific activation pattern (Figure 11A,B). Admira Fusion dust markedly increased the phospho-p38/p38 ratio, showing a consistent elevation across biological replicates (mean fold-change ~1.8–2.0 relative to control). In contrast, Ceram.x^®^ Spectra ST dust maintained phospho-p38/p38 ratios at or slightly below baseline, indicating no detectable activation of the p38-MAPK pathway under this particulate condition.

Because phosphorylation represents the catalytically active kinase state, these data suggest selective activation of p38 in Admira Fusion-treated cells, whereas Ceram.x^®^ Spectra ST does not induce measurable MAPK engagement despite comparable total p38 levels. Thus, the phospho-p38/p38 pattern highlights a material-specific divergence in MAPK signaling, consistent with engagement of stress-responsive pathways. Thus, the phospho-p38/p38 pattern highlights a material-specific divergence in MAPK signaling. To assess whether this activation profile corresponded to functional downstream output, we next quantified IL-8 secretion.

### 3.9. Divergent IL-8/CXCL8 Secretion in Response to Particulate and Eluate Exposure

**Figure 12:**
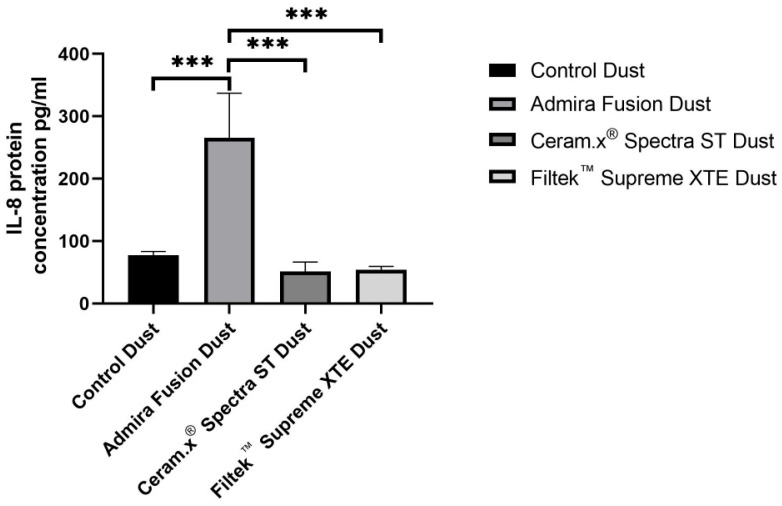
IL-8 secretion by gingival keratinocytes after composite dust exposure. Keratinocytes were exposed for 24 h to control dust or to dusts generated from the composites Admira Fusion, Ceram.x^®^ Spectra ST and Filtek™ Supreme XTE. IL-8 protein concentrations in cell supernatants were measured by ELISA. Bars show mean ± SD from three independent experiments. Statistical differences between indicated conditions were assessed by one-way ANOVA followed by appropriate post hoc testing; *** *p* < 0.001.

As a downstream functional readout of the observed material-dependent differences in SIRT1, NF-κB, and p38 signaling, we next quantified IL-8 (CXCL8) secretion in HGKs exposed to composite dusts. ELISA analysis revealed that Admira Fusion dust increased IL-8 release by approximately 3–4-fold relative to untreated controls (*p* < 0.001; Figure 12), whereas Filtek™ Supreme XTE dust did not significantly increase IL-8 (remaining at baseline levels, similar to control) and Ceram.x^®^ Spectra ST dust likewise failed to elevate IL-8 secretion above baseline (Figure 12).

These observations identify a distinct IL-8 secretion profile for particulate exposure and provide a functional readout linking upstream SIRT1, NF-κB, and p38 signaling events to cytokine output.

## 4. Discussion

Resin-based composites are indispensable in contemporary restorative dentistry, yet despite their extensive clinical use, the cellular and molecular consequences of composite-derived particulate exposure at the gingival interface remain insufficiently characterized. Previous work has focused on chemical eluates released from dental restorative materials, reporting associations with cytotoxicity, oxidative stress, and altered barrier function [25,26,27]. However, particulate dust generated during finishing and polishing procedures represents a separate and understudied exposure scenario, particularly relevant because particles can directly contact epithelial surfaces. To explore how particulate and eluate fractions of different composite categories modulate epithelial responses, we deliberately selected three widely used restorative materials that exemplify distinct resin and filler categories—Filtek™ Supreme XTE, Ceram.x^®^ Spectra ST, and Admira Fusion. By comparing these representatives of nanofilled, nano-hybrid methacrylate, and Ormocer composites, we show that particulate dusts trigger stronger and more diverse perturbations than their corresponding eluates. Moreover, each composite’s distinctive chemistry and filler morphology give rise to material-specific signatures in particle morphometry, epithelial function, signaling pathways and inflammatory output, underscoring the relevance of resin and filler composition in determining biological responses. One of the central new approaches of this work lies in integrating morphometric analysis of the dust particle clustering with downstream biological responses. SEM imaging and quantitative morphometry revealed that Admira Fusion dust yielded relatively large clusters with low circularity and higher aspect ratios, indicating irregular, elongated fragments; Filtek™ Supreme XTE dust generated the smallest and most spherical particles (highest circularity and solidity); Ceram.x^®^ Spectra ST particles were intermediate in size and shape. These differences translated into striking variations in surface coverage: Filtek™ Supreme XTE dust produced roughly three times as many particles per square millimeter of cell surface as Admira Fusion, with Ceram.x^®^ Spectra ST again intermediate, likely reflecting differences in filler size distribution and nanoparticle content among the materials.

The morphometric analysis provided here illustrates how the geometry of the dust particles reflects the distinct filler architecture of each composite. To be more precise, Filtek™ Supreme XTE is a true nanocomposite in which all fillers are engineered nanoparticles. It contains non-agglomerated silica and zirconia plus aggregated nanoclusters ranging from 0.6–10 μm. These nanofillers and clusters are fused rather than ground, and they shear during abrasion at a rate similar to the surrounding resin, which explains why dust from this material formed the smallest and most spherical particles in our morphometry. Ceram.x^®^ Spectra ST is a nano-hybrid composite that blends pre-polymerized SphereTEC^®^ granules with non-agglomerated barium glass and ytterbium fluoride, and its methacrylate matrix contains highly dispersed polysiloxane nanoparticles. These larger, spherical granules, combined with sub-micron fillers, account for the intermediate particle size and shape we observed. On the other hand, Admira Fusion is an ormocer. Its resin matrix and fillers are both based on silicon-oxide. The material contains glass-ceramic fillers (~1 μm) and nanofillers but is completely free of conventional monomers. The inorganic SiO_2_ backbone and micro-nano hybrid filler system result in larger, irregular and elongated fragments, consistent with the low circularity and higher aspect ratio measured in our analysis.

Such disparities are likely to modulate how keratinocytes perceive and respond to the particles. Smaller and more spherical particles may increase the extent of cell–particle contact and, depending on surface properties and aggregation state, can influence cellular internalization and downstream stress/inflammatory responses [28]. Indeed, composite grinding has been shown to release a high fraction of nano-sized particles, median diameters 15–35 nm and even as low as 38–70 nm [18,29], and >80% of composite dust particles have minimum Feret diameters < 1 μm [18]. Inhalation of such ultrafine dust can deposit particles in the alveolar region, where they are difficult to clear, potentially causing chronic inflammation and fibrosis [8]. On keratinocytes, the high particle density of Filtek™ Supreme XTE dust may similarly impede normal cell–substrate interactions and lead to detachment or anoikis, consistent with observations that nano-composite dust suppresses extracellular matrix deposition and cell adhesion [8]. By contrast, larger Admira Fusion dust clusters appear to behave as discrete mechanical stimuli rather than a continuous coating, possibly triggering integrin-mediated mechanotransduction pathways. Mechanical strain and focal adhesion engagement can activate ERK/MAPK signaling and promote IL-8 release [8], which may explain the transient inflammatory responses observed with Admira Fusion dust.

The shape metrics also imply differing bioreactivity. The irregular, high-aspect-ratio particles in Admira Fusion dust may create protruding edges that physically stress membranes, whereas the compact Filtek™ Supreme XTE particles are more readily internalized or engulfed. Alveolar macrophage studies support this concept: nano-composite dust particles are phagocytosed by macrophages and can accumulate in cytoplasm without immediate cytotoxicity, but persistent particle loading may eventually induce oxidative stress and cytokine production [29]. Extrapolating to gingival keratinocytes, frequent exposure to ultrafine Filtek™ Supreme XTE dust could overwhelm cellular clearance mechanisms and exacerbate chronic inflammation. Conversely, ormocer-based materials like Admira Fusion contain less methacrylate monomer and have been reported to release cytotoxic components [30]. Their larger particle size may therefore induce acute but manageable inflammation without significant long-term toxicity. To our knowledge, this represents the first direct demonstration of a morphometric–signaling–cytokine axis in gingival epithelial responses to dental composite dust.

These findings indicate that biological responses cannot be attributed to resin chemistry alone. Instead, they likely arise from the combined influence of resin matrix chemistry, filler architecture, particle size, projected particle burden, surface morphology, aggregation state, and sedimentation behavior. Although Admira Fusion particles were larger and more irregular, Ceram.x^®^ Spectra ST showed the highest estimated projected contact burden based on SEM-derived particle area and density. Therefore, the IL-8 response observed for Admira Fusion cannot be explained solely by total projected surface coverage. Nevertheless, because true particle surface area, sedimentation kinetics, density, surface charge, and nanoscale chemical reactivity were not directly measured, these parameters remain important confounders and should be investigated in future work.

Beyond the inherent properties of the particles themselves, our study demonstrates that fundamental epithelial functions are impaired differently by particulate exposure compared to eluate exposure. In our experiments, dust from Filtek™ Supreme XTE and Ceram.x^®^ Spectra ST composites markedly suppressed keratinocyte proliferation, whereas the corresponding eluates caused only a moderate (Filtek XTE) or no significant (Ceram.x ST) decline in CI, underscoring the far stronger impact of particulate exposure. This pattern is consistent with observations in pulmonary epithelial systems, where inhalable particulate fractions induce more pronounced cytotoxic effects than their solubilized counterparts [31]. HGKs similarly displayed heightened sensitivity to composite-derived particulates, reflected by reduced proliferative dynamics, and altered differentiation profiles. The fact that Admira Fusion dust induced only a transient CI reduction despite its structurally complex particles, whereas Filtek™ Supreme XTE dust—with a simpler particulate morphology—produced the strongest suppression of differentiation markers, indicates that particle architecture alone does not predict biological potency. Instead, the data suggest that composite-specific resin chemistry and filler composition interact with particulate features to shape epithelial responses. In addition to proliferation, keratinocyte differentiation, a central determinant of gingival resilience, was also compromised primarily under particulate exposure. Filtek™ Supreme XTE and Ceram.x^®^ Spectra ST dust markedly reduced early differentiation markers KRT1 and KRT10 and, to a lesser extent, late markers FLG and IVL. This is consistent with previous observations that methacrylate-based materials interfere with epithelial homeostasis, including proliferation and differentiation [32,33]. However, our data extend these findings by demonstrating that particulate fractions are more potent disruptors of differentiation programs than eluates, indicating that physical particle properties contribute to this phenotype through mechanisms not solely explained by soluble components. Admira Fusion, as an Ormocer-based composite, exhibited minimal impairment of differentiation, suggesting that network chemistry and polymerization behavior may modulate dust bioactivity—potentially by reducing the fraction of unreacted, extractable components and thereby limiting material release. In this context, Filtek™ Supreme XTE emerges as the strongest inducer of an ERK-suppressed, ECM-compromised phenotype, whereas Admira Fusion maintains FN1 despite its higher morphometric complexity. This divergence further emphasizes that structural particle attributes and resin chemistry jointly determine epithelial outcomes.

These differentiation defects were closely mirrored by alterations in fibronectin (FN1), an essential ECM component orchestrating adhesion, migration, and wound closure. Filtek™ Supreme XTE and Ceram.x^®^ Spectra ST dusts strongly suppressed FN1 protein levels, an effect consistent with the well-established sensitivity of ECM assembly to differentiation status and stress-activated pathways [34]. In contrast, Admira Fusion preserved FN1 expression despite comparable particulate complexity, underscoring chemistry-dependent divergence in ECM regulation. Given that FN1 expression is tightly controlled by ERK signaling [15], our pharmacological MEK-inhibition experiments reinforce that ERK activity contributes to maintaining FN1 abundance under the present conditions in gingival keratinocytes. The fact that Filtek™ Supreme XTE and Ceram.x^®^ Spectra ST suppressed ERK together with FN1, whereas Admira Fusion activated ERK while preserving FN1, establishes a clear and coherent, composite-specific ERK–FN1 axis. This is highly consistent with work showing that fibronectin–integrin engagement activates ERK/MAPK pathways and supports downstream NF-κB and chemokine programs, including IL-8 [35,36,37,38,39,40].

Beyond ECM control, our data identify a previously underexplored stress-associated readout in gingival epithelial responses to composite-derived particles. SIRT1 is a conserved NAD^+^-dependent deacetylase involved in chromatin stability, oxidative stress adaptation, and inflammatory regulation [41,42]. In inflammatory signaling, SIRT1 can restrain NF-κB activity through deacetylation of p65/RelA [42,43], Prior work has shown that phosphorylation of SIRT1 at Ser682 can restrict SIRT1 activity by disrupting its interaction with the co-activator AROS [12,16]. In this context, the reduction in SIRT1-S682 observed after particulate exposure may reflect a shift away from an inhibitory SIRT1 modification. However, this interpretation remains inferential. We did not measure SIRT1 deacetylase activity, p65 acetylation, NAD^+^ availability, or downstream SIRT1 substrate activity, and we did not manipulate SIRT1 expression or Ser682 phosphorylation. Therefore, SIRT1-S682 should be interpreted here as a correlative marker of stress-associated nuclear regulation, not as a functionally validated regulatory switch. We use the term “permissive” only to describe a potential nuclear regulatory context in which transcription factor availability may be altered; it does not imply that SIRT1-S682 reduction is sufficient to induce NF-κB activation or IL-8 secretion. Despite this shared reduction in SIRT1-S682, downstream inflammatory outputs differed among materials. This distinction is important because it shows that SIRT1-S682 reduction was not sufficient to generate an IL-8 response. Filtek™ Supreme XTE and Ceram.x^®^ Spectra ST reduced SIRT1-S682 but did not induce IL-8 secretion above baseline. In contrast, Admira Fusion combined SIRT1-S682 reduction with preserved FN1, ERK activation, p38 activation, and comparatively maintained NF-κB p65 Ser536 phosphorylation. Thus, the observed inflammatory phenotype is best interpreted as part of a broader material-associated signaling pattern rather than as a direct consequence of SIRT1-S682 modulation alone. Total NF-κB p65 protein abundance showed biological variability but no consistent or statistically significant material-dependent change. Therefore, total p65 was treated as a baseline abundance readout, while NF-κB activation was evaluated using localization, Ser536 phosphorylation, and IL-8 secretion. Within this focused comparison, a distinct divergence emerged. Both Admira Fusion and Ceram.x^®^ Spectra ST dust increased the nuclear-to-cytoplasmic ratio of NF-κB p65, yet only Admira Fusion markedly elevated nuclear p65 signal intensity and induced IL-8 secretion. Ceram.x^®^ Spectra ST, despite achieving similar N/C ratios, failed to maintain p65 Ser536 phosphorylation and therefore generated a predominantly transcriptionally inert nuclear NF-κB pool. This pattern is physiologically coherent, as phosphorylation of p65 at Ser536 is required for transcription of IL-8 and other NF-κB target genes, whereas its absence prevents promoter engagement even in the presence of nuclear NF-κB [44]. The marked Ser536 dephosphorylation under Ceram.x^®^ Spectra ST, together with ERK and FN1 suppression, demonstrates that SIRT1-S682 reduction alone does not license NF-κB activation. Instead, only Admira Fusion provides the necessary upstream ERK–FN1 stimulus to render nuclear NF-κB transcriptionally competent, consistent with fibronectin–MAPK pathways known to activate NF-κB-dependent chemokine expression, including IL-8 [35,36,37,38,39,40]. Admira Fusion uniquely fulfilled these mechanistic prerequisites. This distinct activation pattern underscores that the specific resin–filler architecture of ormocer materials can potentiate inflammatory signaling, and it highlights the need to scrutinize composite composition when evaluating cytocompatibility. Its relative preservation of NF-κB Ser536 phosphorylation and robust p38-MAPK activation, a kinase essential for IL-8 transcription [45], sharply contrasted with Ceram.x^®^ Spectra ST, which showed marked Ser536 dephosphorylation and lacked p38 activation.

The absence of p38 activation in Ceram.x^®^ Spectra ST-treated cells precisely paralleled their inactive NF-κB-Ser536 profile, reinforcing a bifurcated signaling architecture in which p38 serves as the decisive upstream discriminator of inflammatory potential. The downstream consequence was unequivocal; only Admira Fusion dust triggered IL-8 secretion, whereas Ceram.x^®^ Spectra ST dust maintained IL-8 at baseline despite comparable NF-κB translocation. These data confirm that nuclear accumulation of NF-κB is insufficient to drive inflammatory output. Instead, coordinated activation of both NF-κB Ser536 and p38 is required to unlock IL-8 transcription. This convergence of ERK, FN1, NF-κB-Ser536, and p38 activation in Admira Fusion, absent in Ceram.x^®^ Spectra ST, demonstrates that the inflammatory phenotype is driven primarily by a composite-specific ERK/FN1/MAPK signaling module rather than by differences in SIRT1-S682 alone.

Integrating these observations, we propose a working model rather than a proven causal pathway. First, particulate exposure universally reduces SIRT1-S682, establishing a permissive nuclear context that modulates transcription factor availability. Second, only materials capable of assembling an ERK- and FN1-linked stress module with concomitant p38 activation, exemplified by Admira Fusion, provide sufficient upstream input to convert this permissive background into a transcriptionally competent NF-κB/IL-8 response, fully consistent with fibronectin/MAPK pathways known to drive NF-κB-dependent chemokine expression [37]. Ceram.x^®^ Spectra ST, despite comparable SIRT1-S682 reduction, fails to engage these ERK- and p38-dependent signaling arms required for NF-κB transcriptional activation, remaining locked in a non-inflammatory state. Filtek™ Supreme XTE, with insufficient NF-κB nuclear enrichment, remains outside this decision architecture altogether. These findings indicate that neither particle morphology nor material chemistry alone is sufficient to explain the observed inflammatory divergence. Instead, the data point to a multifactorial interplay in which compositional differences and ultrastructural particle features jointly shape the signaling trajectories that determine whether NF-κB becomes transcriptionally engaged. U0126 supports a role for ERK in maintaining FN1 expression, but the current study did not include FN1 knockdown, p38 inhibition, NF-κB blockade, or rescue experiments. Therefore, the proposed ERK–FN1–p38–NF-κB sequence should be interpreted as a correlative signaling framework that requires functional validation.

Taken together, these findings establish a coherent, composite-specific inflammatory signature (i) a SIRT1-permissive but ERK-driven inflammatory activation pathway, observed for Admira Fusion under the present in vitro conditions, and (ii) a SIRT1-permissive but ERK-suppressed, transcriptionally inert state characteristic of Ceram.x^®^ Spectra ST. This dual-mechanism model provides new conceptual clarity in composite biocompatibility research and may reconcile previously conflicting observations regarding particulate cytoreactivity. A particularly notable implication is that Ormocer-based materials, often marketed for enhanced biocompatibility [46], can exhibit particle-specific inflammatory liabilities despite favorable eluate profiles. Conversely, the ECM-disruptive effects observed for Filtek™ Supreme XTE and Ceram.x^®^ Spectra ST highlight that attenuation of inflammation does not guarantee preserved tissue integrity, emphasizing the need to assess ECM stability alongside cytotoxicity in peri-restorative biocompatibility evaluations.

Although our in vitro model reveals distinct signaling patterns and particle morphologies among the composites, it lacks key elements of the oral environment such as immune–epithelial crosstalk, microbial biofilms and mechanical loading. The identified signaling architecture (ERK, SIRT1, NF-κB, p38) therefore provides mechanistic insights rather than direct clinical predictions. Particle deposition during clinical polishing is intermittent and of short duration [3]. Our acute exposure paradigm approximates certain aspects of these episodes but does not replicate long-term wear or repeated exposure cycles. Further, clinical studies comparing ormocer-based composites with conventional methacrylates have not identified clear advantages of ormocers; if anything, some analyses report a higher rate of post-operative sensitivity for early ormocer formulations [47]. Thus, while our results suggest material-specific in vitro inflammatory responses, we do not claim clinical superiority of one composite over another. Future work integrating primary cells, organotypic cultures and long-term exposure models will be required to determine whether these in vitro signatures translate to peri-restorative tissue outcomes in vivo. Beyond, the molecular basis for the differential Admira Fusion response remains unresolved. Admira Fusion differs from the methacrylate-based composites not only in matrix architecture but also in filler composition, particle morphology, and likely surface reactivity. Its silicon-oxide-based ormocer network and larger irregular fragments may alter cell–particle contact, integrin engagement, and mechanotransduction, thereby indirectly influencing ERK/FN1 signaling. However, we did not identify specific leachable compounds, surface functional groups, charge properties, or nanoscale chemical features responsible for FN1 preservation or SIRT1-S682 modulation. Importantly, our data do not suggest a direct interaction between Admira Fusion particles and the SIRT1 Ser682 regulatory site. Rather, SIRT1-S682 changes are interpreted as downstream indicators of altered cellular stress regulation. Future studies combining LC–MS/MS leachables profiling, zeta-potential measurements, nano-IR, Raman spectroscopy, EDX mapping, and targeted perturbation of mechanotransduction pathways will be required to define the molecular determinants of this response.

In summary, this study provides the most integrated analysis to date of how composite-derived particulate and chemical exposures shape gingival epithelial signaling. By coupling particle cluster morphometry with multi-level molecular readouts, we identify reduced SIRT1-S682 as a shared particulate exposure-associated stress readout and show that IL-8 secretion occurred only under conditions accompanied by ERK/FN1 preservation, p38 activation, and comparatively maintained NF-κB p65 Ser536 phosphorylation. These findings support an associative signaling framework, while direct functional studies will be required to determine whether SIRT1-S682 actively contributes to the inflammatory phenotype. These insights may inform, rather than define, the evaluation of next-generation restorative materials and underscore that particulate fractions could influence peri-restorative tissue health, but confirmation in clinically relevant models is needed [3].

## 5. Conclusions

Within the limitations of our in vitro model, composite-derived particulate dust exerted stronger and more diverse effects on gingival keratinocytes than chemical eluates, reflecting material-specific differences in physicochemical properties and downstream signaling. In these immortalized keratinocytes, Filtek™ Supreme XTE and Ceram.x^®^ Spectra ST dusts suppressed extracellular signal-regulated kinase (ERK) activity, reduced fibronectin (FN1) abundance and decreased nuclear SIRT1-S682. In contrast, Admira Fusion dust preserved FN1, activated ERK and p38, and led to NF-κB Ser536 phosphorylation and IL-8 induction. These findings support a distinct in vitro inflammatory response pattern associated with Admira Fusion dust, rather than a universally “unique” program. Extrapolation to the in vivo gingival environment should therefore be approached with caution, and further studies using primary cells and tissue models are warranted to determine how these in vitro signatures translate to clinical settings. This mechanistic framework underscores the multifactorial nature of composite bioactivity and highlights in the context of particle cluster metrics, a continuum from few large, irregular particles (Admira Fusion) to numerous small, spherical particles (Filtek™ Supreme XTE), with Ceram.x^®^ Spectra ST occupying an intermediate niche. These physical differences correlate with distinct biological outcomes—mechanical stimulation and transient inflammatory signaling for larger particles versus widespread surface coverage and potential internalization for nanoscale dust. Recognizing how composite formulation influences both particle morphology and cell behavior is essential when evaluating the safety and biocompatibility of restorative materials.

## Figures and Tables

**Table 1:** Particle cluster morphometrics of composite dust on keratinocyte surfaces (mean ± SD) for three composite materials (Admira Fusion, Ceram.x^®^ Spectra ST, Filtek™ Supreme XTE). Statistical significance was determined by one-way ANOVA followed by Tukey’s post hoc test. * *p* < 0.05 vs. the preceding group; *** *p* < 0.001 vs. Admira Fusion.

Metric	Admira Fusion	Ceram.x^®^ Spectra ST	Filtek™ Supreme XTE
**Particle area (μm^2^)**	1.6 ± 3.5 ***	1.0 ± 2.0 *	0.4 ± 1.5
**Eq. circ. diameter (μm)**	1.4 ± 1.5 ***	1.1 ± 1.0 *	0.7 ± 0.8
**Circularity (a.u.)**	0.72 ± 0.15	0.80 ± 0.10 *	0.88 ± 0.07 ***
**Solidity (a.u.)**	0.92 ± 0.08	0.96 ± 0.04 *	0.98 ± 0.02 ***
**Aspect ratio (major/minor)**	1.8 ± 0.5 ***	1.4 ± 0.3 *	1.2 ± 0.2
**Particle density (#/mm^2^)**	5.0 × 10^4^ ± 5 × 10^3^	1.0 × 10^5^ ± 1 × 10^4^ *	1.5 × 10^5^ ± 2 × 10^4^ ***

## Data Availability

The original contributions presented in this study are included in the article material. Further inquiries can be directed to the corresponding author(s).

## Supplementary Materials

The following supporting information can be downloaded at: https://www.mdpi.com/article/10.3390/jfb17060264/s1.

## Abbreviations

The following abbreviations are used in this manuscript:

**Abbreviation**	**Full Term**
ANOVA	Analysis of variance
BCA	Bicinchoninic acid
BSA	Bovine serum albumin
CI	Cell index
CXCL8	C-X-C motif chemokine ligand 8
DAPI	4′,6-diamidino-2-phenylindole
DLS	Dynamic light scattering
DMSO	Dimethyl sulfoxide
DPBS	Dulbecco’s phosphate-buffered saline
ECM	Extracellular matrix
EDX	Energy-dispersive X-ray
ELISA	Enzyme-linked immunosorbent assay
ERK1/2	Extracellular signal-regulated kinase 1/2
FLG	Filaggrin
FN1	Fibronectin
HGKs	Human gingival keratinocytes
HSD	Honestly significant difference
IIF	Indirect immunofluorescence
IL-8	Interleukin-8
IVL	Involucrin
KGM2	Keratinocyte growth medium 2
KRT1	Keratin 1
KRT10	Keratin 10
LSD	Least significant difference
MAPK	Mitogen-activated protein kinase
MEK1/2	Mitogen-activated protein kinase kinase 1/2
nano-IR	Nanoscale infrared spectroscopy
N/C	Nuclear-to-cytoplasmic ratio
NF-κB	Nuclear factor κB
p38-MAPK	p38 mitogen-activated protein kinase
PCR	Polymerase chain reaction
PVDF	Polyvinylidene fluoride
qPCR	Quantitative real-time PCR
RelA	NF-κB p65 subunit
RIPA	Radioimmunoprecipitation assay buffer
RPL13A	Ribosomal protein L13a
RTCA	Real-time cell analysis
SD	Standard deviation
SIRT1	Sirtuin 1
UBC	Ubiquitin C
VIM	Vimentin
