# Supplementary material for: A Dusty Affair: SIRT1-S682 Modulation Orchestrates ERK–FN1–p38–NF-κB Signaling and Composite-Dependent IL-8 Responses in Gingival Keratinocytes Exposed to Dental Dust and Eluates
Source: J Funct Biomater. 2026 Jun 1;17(6):264. doi: 10.3390/jfb17060264 (PMC13301396; doi:10.3390/jfb17060264)
Supplement: Supplementary file 1 [file jfb-17-00264-s001.zip › jfb-4226107-supplementary.pdf]

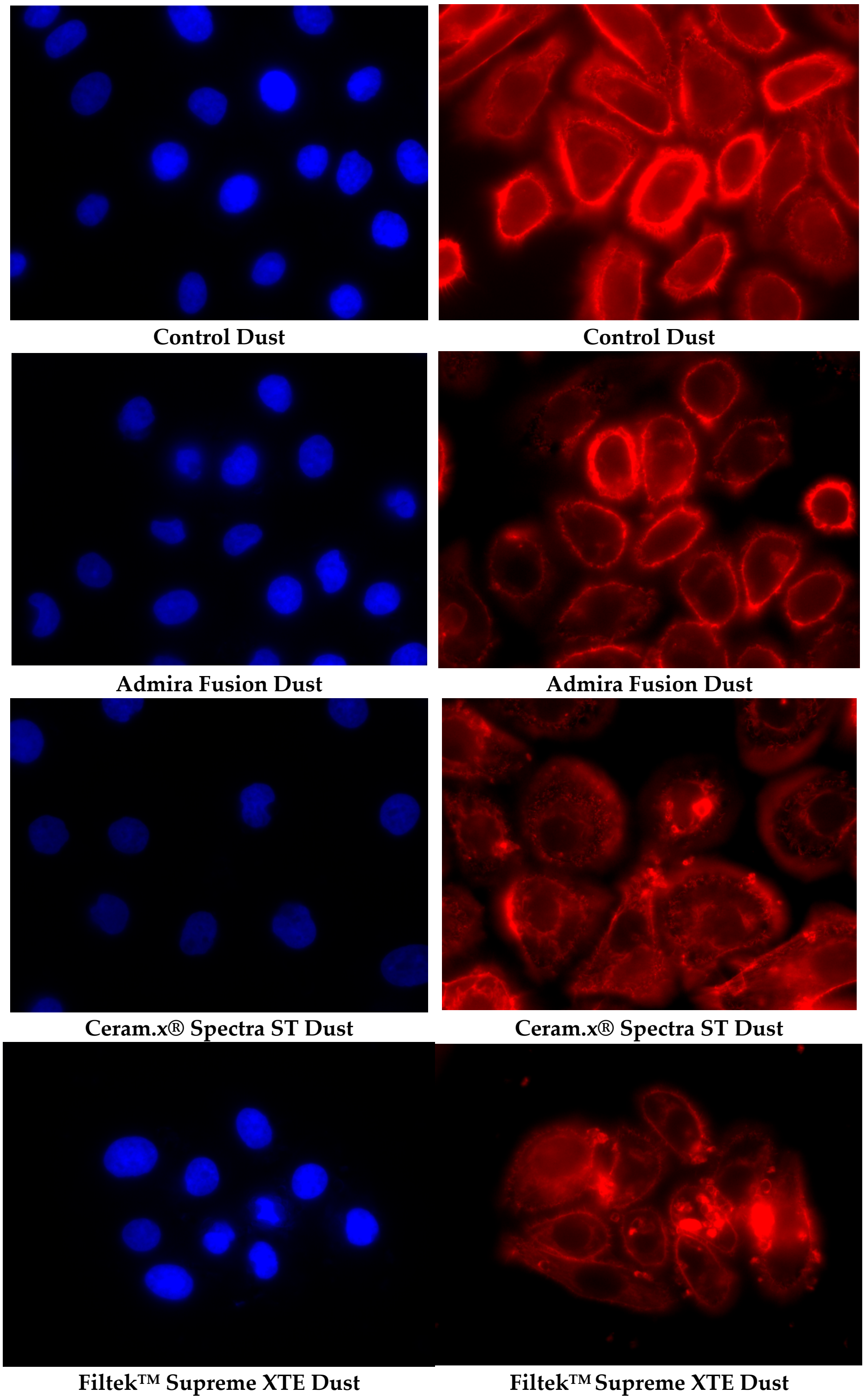

Supplementary Figure S1. Supplementary images for Figure 8A, localization and quantification of SIRT1 Ser682 phosphorylation following dust exposure on gingival keratinocytes, showing the immunofluorescence channels not yet shown in an unmerged state. The DAPI staining shows the cell nucleus in blue, and the phalloidin staining shows F-actin in red to visualize the entire cell. The exposure was conducted over a 24-hour period using Dust produced from Admira Fusion, Ceram.x® Spectra ST or Filtek™ Supreme XTE.

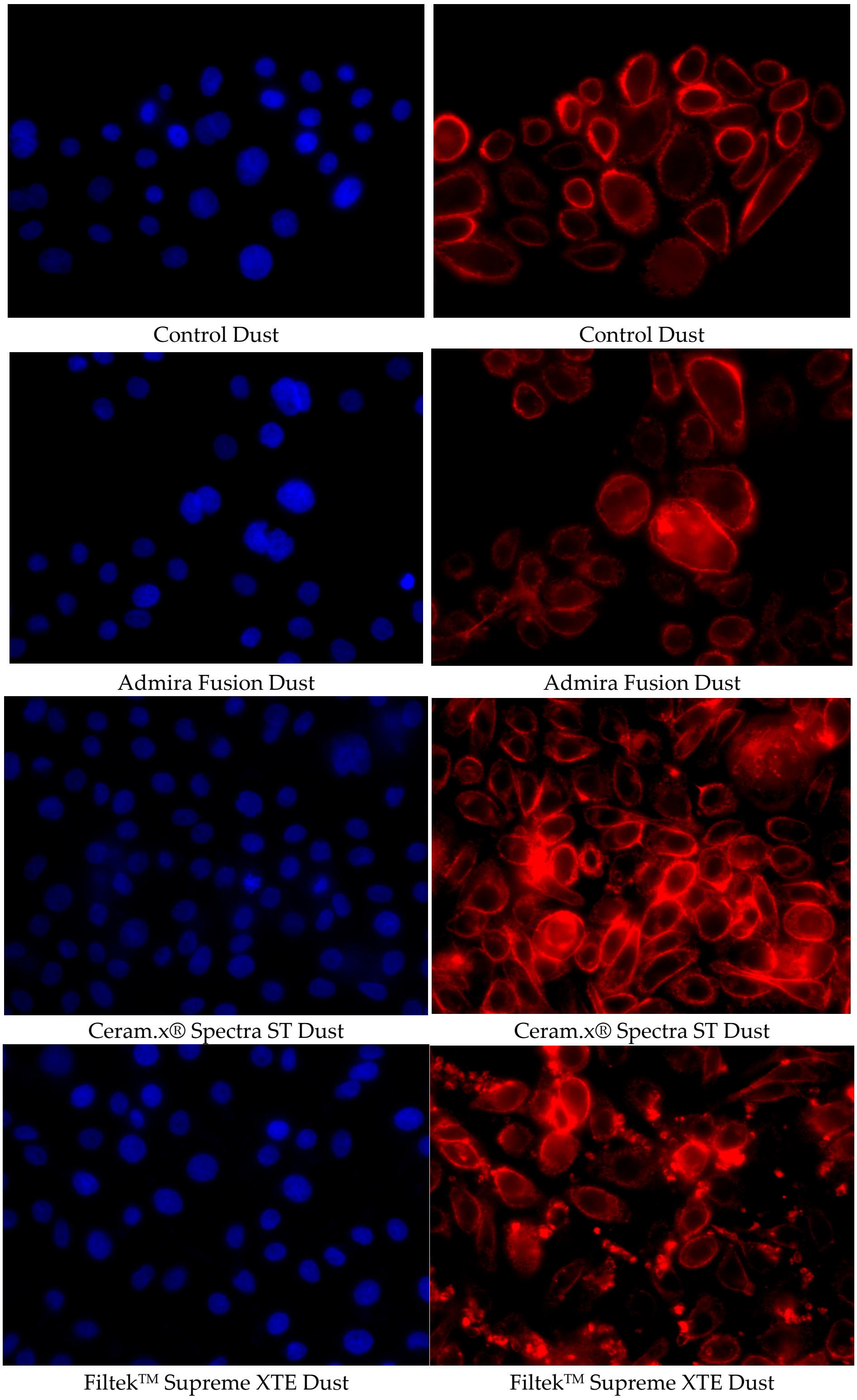

Supplementary Figure S2. Supplementary images for Figure 10A, localization and quantification of phosphorylated NF-κB p65 following dust exposure on gingival keratinocytes, showing the immunofluorescence channels not yet shown in an unmerged state. The DAPI staining shows the cell nucleus in blue, and the phalloidin staining shows F-actin in red to visualize the entire cell. The exposure was conducted over a 24-hour period using Dust produced from Admira Fusion, Ceram.x® Spectra ST or Filtek™ Supreme XTE.
